# Supplementary material for: Soft magnetic hysteresis in a dysprosium amide–alkene complex up to 100 kelvin
Source: Nature. 2025 Jun 25;643(8070):125–9. doi: 10.1038/s41586-025-09138-0 (PMC12221970; doi:10.1038/s41586-025-09138-0)
Supplement: Supplementary file 1 — This file contains Supplementary Methods, Notes, Figs. 1–49 and Tables 1–8. [file 41586_2025_9138_MOESM1_ESM.pdf]

nature portfolio

https://doi.org/10.1038/s41586-025-09138-0

Supplementary information

Soft magnetic hysteresis in a dysprosium amide–alkene complex up to 100 kelvin

In the format provided by the authors and unedited

Supporting Information for:

Soft magnetic hysteresis in a dysprosium amide-alkene complex up to 100 K

Jack Emerson-King[1], Gemma K. Gransbury[1], Benjamin E. Atkinson[1,2], William J. A. Blackmore[1], George F. S. Whitehead[1], Nicholas F. Chilton[1,2] & David P. Mills[1]

[1]*Department of Chemistry, The University of Manchester, Oxford Road, Manchester, M13 9PL, UK.*

[2]*Research School of Chemistry, The Australian National University, Sullivans Creek Road, Canberra, ACT, 2601, Australia.*

Contents

1. Experimental procedures S2
2. NMR spectra S5
3. Powder X-ray diffraction S7
4. IR spectra S9
5. Single crystal X-ray diffraction S12
6. DFT calculations S23
7. CASSCF electronic structure S24
8. Magnetic data S26
9. Ab initio spin dynamics S45

1. Experimental procedures

Synthesis of [Y{N(Si^{i}Pr$_3$)[Si(^{i}Pr)$_2$C(CH$_3$)=CHCH$_3$]}{N(Si^{i}Pr$_3$)(Si^{i}Pr$_2$Et)}][Al{OC(CF$_3$)$_3$}$_4$] (1-Y). A mixture of **2-Y** and HN{S^{i}Pr$_3$)$_2$, (1.700 g, found to contain *ca*. 0.960 g, 1.3 mmol **2-Y** by ^{1}H NMR integration), prepared as described below from YI_3 (2.500 g, 5.00 mmol) and [K{N{S^{i}Pr$_3$)$_2$}] (1.840 g, 5.00 mmol) was treated with [HNEt$_3$][Al{OC(CF$_3$)$_3$}$_4$] (2.890 g, 2.70 mmol) in C_6H_6 (20 mL) for 18 h at 40 °C. All volatiles were removed *in vacuo*, and the residues washed with *n*-hexane (*ca*. 3 × 5 mL). The residues were dissolved in C_6H_5F (~2 mL) and layered under *n*-hexane (*ca*. 20 mL), affording upon diffusion a pale-yellow oil beneath colourless crystals of [HNEt$_3$][Al{OC(CF$_3$)$_3$}$_4$]. The oil was decanted into a new flask and the crystallisation process repeated nine times, at which point no additional [HNEt$_3$][Al{OC(CF$_3$)$_3$}$_4$] was observed in the residual oil by ^{1}H NMR spectroscopy. The final supernatant was decanted and **1-Y** was recrystallised by slow evaporation of residual solvent at ambient pressure. The crystalline material was triturated with excess *n*-hexane and dried *in vacuo*. Yield: 0.188 g, 109.9 mmol, 8% taking **2-Y** as the limiting reagent. ^{1}H NMR (400.07 MHz, C_6H_5F): δ 7.01–6.95 (m, 1H, SiC(CH$_3$)C<u>*H*</u>CH$_3$), 1.96 (s, 3H, SiC(C<u>*H*</u>$_3$)CHCH$_3$), 1.90 (d, $^3J_{HH}$ = 5.8 Hz, SiC(CH$_3$)CHC<u>*H*</u>$_3$), 1.25 (t, $^3J_{HH}$ = 7.0 Hz, 3H, SiCH$_2$C<u>*H*</u>$_3$,), 1.27–0.66 (multiple coincident ^{i}Pr resonances & SiC<u>*H*</u>$_2$CH$_3$, ~ 72H). ^{13}C{^{1}H} DEPTQ NMR (100.60 MHz, C_6H_5F): δ 163.37 (s, Si<u>*C*</u>(CH$_3$)CHCH$_3$), 149.81 (d, $^1J_{YC}$ = 1.5 Hz, SiC(CH$_3$)<u>*C*</u>HCH$_3$), 122.17 (q, $^1J_{FC}$ = 292.9 Hz, C(<u>*C*</u>F$_3$)$_3$), 19.97 (s, ^{i}Pr), 19.51 (s, SiC(<u>*C*</u>H$_3$)CHCH$_3$, 19.22 (s, ^{i}Pr), 18.18 (s, ^{i}Pr), 18.04 (s, ^{i}Pr), 18.03 (s, ^{i}Pr), 17.86 (s, ^{i}Pr), 17.14 (SiC(CH$_3$)CH<u>*C*</u>H$_3$), 17.03 (d, $^1J_{YC}$ = 2.0 Hz, Si<u>*C*</u>H$_2$CH$_3$), 15.94 (s, ^{i}Pr), 14.16 (s, ^{i}Pr), 9.83 (s, SiCH$_2$<u>*C*</u>H$_3$). The *C*(CF$_3$)$_3$ resonance of the [Al{OC(CF$_3$)$_3$}$_4$]$^-$ anion was not observed in the ^{13}C{^{1}H} NMR spectra of **1-Y**, likely due to quadrupolar broadening by the 100% abundant $I = {}^5/_2$ ^{27}Al nuclei. and coupling to multiple 100% abundant $I = {}^1/_2$

^{19}F nuclei. ^{19}F NMR (376.40 MHz, C_6H_5F): δ –75.15 (s, C*F*$_3$). $^{29}Si\{^1H\}$ DEPT90 NMR (79.48 MHz, C_6H_5F): δ 2.43 (s), –3.91 (s), –7.91 (s), –8.75 (s). Anal. calcd. for $C_{52}H_{82}AlF_{36}N_2O_4Si_4Y$ (1711.41 g mol^{-1}) C, 36.49; H, 4.83; N, 1.64. Found: C, 33.72; H, 4.42; N, 1.50. FTIR (ATR, microcrystalline): $\tilde{\nu}$ = 2954 (m), 2870 (m), 1461 (m), 1349 (m), 1210 (s), 965 (s), 727 (s), 540 (s), 437 (s).

Preparation of 5%Dy@1-Y. A mixture of **1-Y** (81.3 mg, 47.5 µmol) and **1-Dy** (4.5 mg, 2.5 µmol) was dissolved in C_6H_5F (*ca*. 0.5 mL) and layered under *n*-hexane (*ca*. 5 mL), affording on diffusion a pale-yellow oil. The supernatant was decanted and the oil crystallised by slow-evaporation of residual solvent at ambient pressure in an argon-atmosphere glovebox. The crystalline material was triturated with excess *n*-hexane and dried *in vacuo*. Yield: 55.6 mg, 32.4 µmol, 65%. Anal. calcd. for $C_{52}H_{82}AlDy_{0.05}F_{36}N_2O_4Si_4Y_{0.95}$ (1715.09 g mol^{-1}) C, 36.42; H, 4.82; N, 1.63. Found: C, 33.47; H, 4.22; N, 1.43. FTIR (ATR, microcrystalline): $\tilde{\nu}$ = 2954 (m), 2870 (m), 1461 (m), 1349 (m), 1210 (s), 965 (s), 727 (s), 540 (s), 437 (s). ICP-MS: 7% Dy : 93% Y.

Synthesis of [Y{N(Si^{i}Pr$_3$)[Si(^{i}Pr)$_2$C(CH$_3$)CHCH$_2$]}{N(Si^{i}Pr$_3$)(Si^{i}Pr$_2$Et)}] (2-Y). A suspension of YI_3 (1.409 g, 3.00 mmol) and [K{N(Si^{i}Pr$_3$)$_2$}] (3.303 g, 9.00 mmol) in C_6H_6 (20 mL) was stirred in a sealed ampoule at 100 °C for 18 h. The volatiles were then removed *in vacuo*, and the residues extracted into *n*-hexane (*ca*. 3 x 10 mL). The volatiles were again removed to afford a viscous yellow oil containing a mix of **2-Y** and H{N(Si^{i}Pr$_3$)$_2$}; **2-Y** was subsequently isolated by crystallisation from a concentrated HMDSO solution at –35 °C, followed by washing with cold HMDSO (< 5 mL, –35 °C). Yield: 0.334 g, 0.449 mmol, 15%. ^1H NMR (400.07 MHz, C_6D_6): δ 7.10 (ddd, $^3J_{HH}$ = 13.2 Hz, $^3J_{HH}$ = 10.0 Hz, $^1J_{YH}$ = 2.5 Hz, 1H, SiC(CH$_3$)C<u>*H*</u>CH$_2$), 3.45 (dd, $^3J_{HH}$ = 10.0 Hz, $^1J_{YH}$ = 2.4 Hz, 1H, SiC(CH$_3$)CHC<u>*H*</u>$_2$-*cis*-CH), 3.34 (d, $^3J_{HH}$ = 13.6 Hz, 1H, SiC(CH$_3$)CHC<u>*H*</u>$_2$-*trans*-CH), 2.06 (s, 3H, SiC(C<u>*H*</u>$_3$)CHCH$_2$), 1.53 (hept, $^3J_{HH}$ = 7.3 Hz,

1H, ^{i}Pr), 1.38 – 1.23 (several coincident ^{i}Pr resonances, 29H), 1.47 – 1.05 (several coincident ^{i}Pr resonances & SiCH$_2$C$\underline{H}_3$ (1.17, *HMBC*), 40H), 1.05 – 0.94 (two coincident ^{i}Pr hept resonances, 2H), 0.90 (td, $^3J_{HH}$ = 7.0 Hz, J_{YH} = 3.1 Hz, 2H, SiC$\underline{H}_2$CH$_3$), 0.86 (hept, $^3J_{HH}$ = 7.3 Hz, 2H, ^{i}Pr). ^{13}C{^{1}H} DEPTQ NMR (100.60 MHz, C_6D_6): δ 161.43 (d, $^1J_{YC}$ = 2.4 Hz, SiC(CH$_3$)$\underline{C}$HCH$_2$), 105.96 (s, Si$\underline{C}$(CH$_3$)CHCH$_2$, 73.72 (d, $^1J_{YC}$ = 6.8 Hz, SiC(CH$_3$)CH$\underline{C}$H$_2$), 27.07 (s, SiC($\underline{C}$H$_3$)CHCH$_2$), 21.60 (s, ^{i}Pr), 20.58 (s, ^{i}Pr), 20.44 (s, ^{i}Pr), 19.93 (s, ^{i}Pr), 19.90 (s, ^{i}Pr), 19.88 (s, ^{i}Pr), 19.41 (s, ^{i}Pr), 19.17 (s, ^{i}Pr), 19.13 (s, ^{i}Pr), 19.05 (s, ^{i}Pr), 19.02 (s, ^{i}Pr), 18.91 (s, ^{i}Pr), 18.84 (s, ^{i}Pr), 18.16 (s, ^{i}Pr), 16.77 (s, ^{i}Pr), 16.02 (s, ^{i}Pr), 15.87 (s, ^{i}Pr), 15.33 (s, ^{i}Pr), 14.98 (s, ^{i}Pr), 13.71 (s, ^{i}Pr), 9.17 (s, SiCH$_2\underline{C}$H$_3$), 7.80 (d, J_{YC} = 2.9 Hz, Si$\underline{C}$H$_2$CH$_3$). ^{29}Si{^{1}H} DEPT90 NMR (79.48 MHz, C_6D_6): δ –2.72 (s), –3.17 (s), –7.89 (s), –15.92 (s). Anal. calcd. for $C_{36}H_{81}N_2Si_4Y$ (743.30 g mol^{-1}) C, 58.17; H, 10.98; N, 3.77. Found: C, 55.74; H, 10.89; N, 3.54. FTIR (ATR, microcrystalline): $\tilde{\nu}$ = 2938 (s), 2857 (s), 2757 (w), 2705 (w), 1515 (w), 1461 (s), 938 (s), 880 (s), 688 (s), 652 (s).

2. NMR spectra

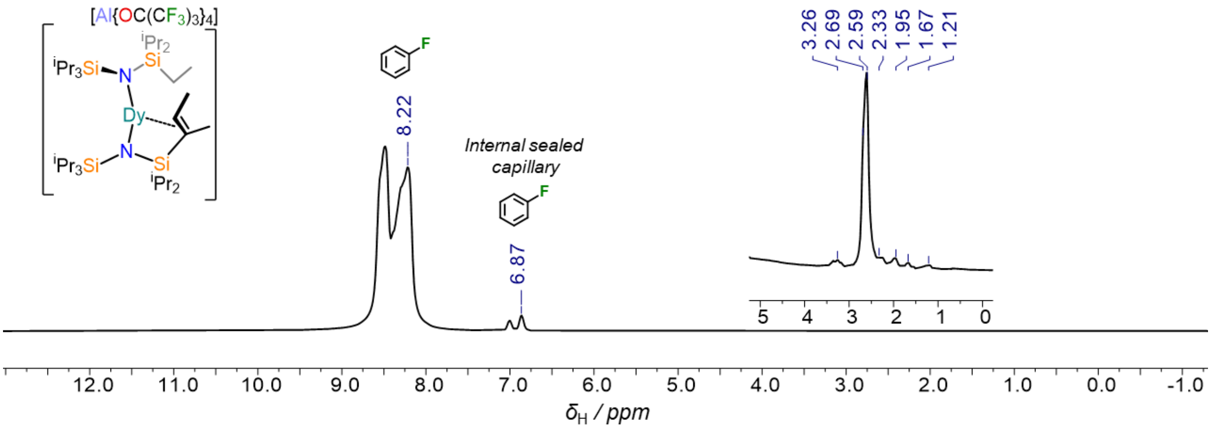

Fig. S1 | ^{1}H NMR spectrum (400.07 MHz, C_6H_5F, 298 K) of 1-Dy.

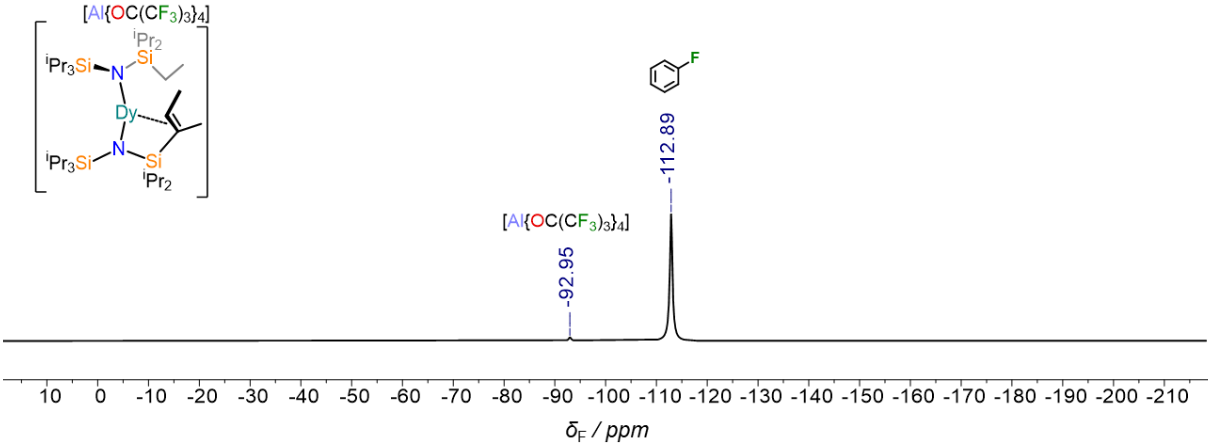

Fig. S2 | ^{19}F NMR spectrum (376.40 MHz, C_6H_5F, 298 K) of 1-Dy.

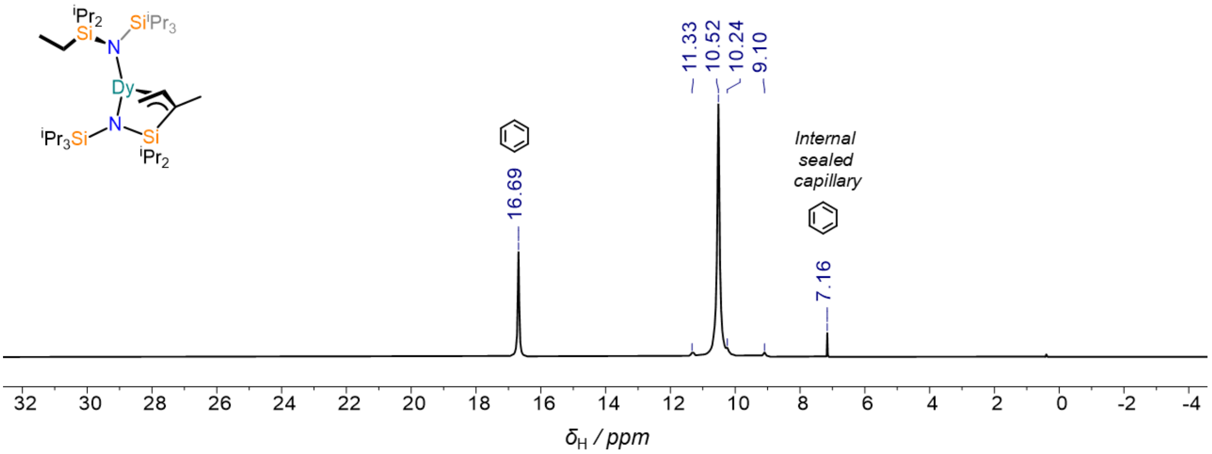

Fig. S3 | ^{1}H NMR spectrum (400.07 MHz, C_6H_5F, 298 K) of 2-Dy.

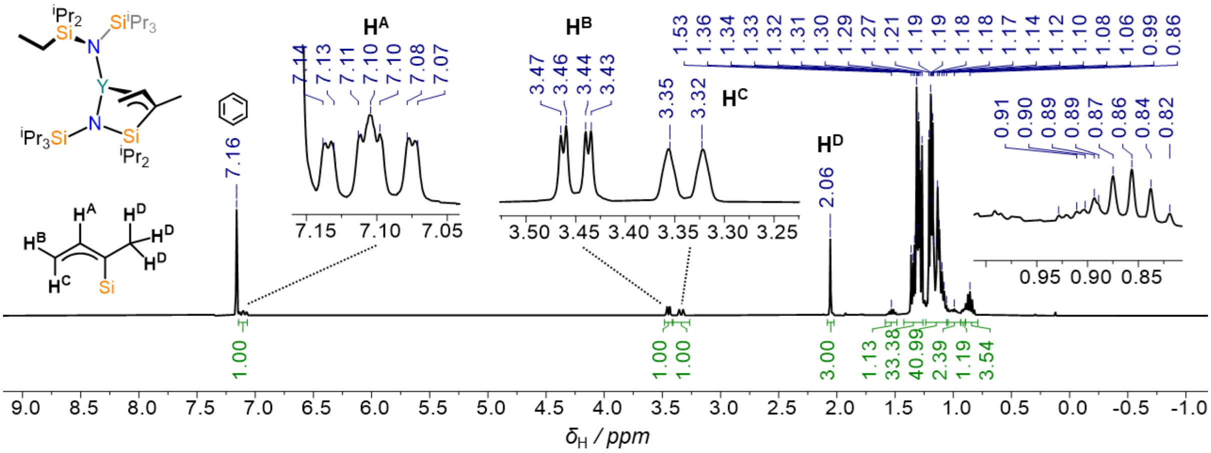

Fig. S4 | ^{1}H NMR spectrum (400.07 MHz, C_6D_6, 298 K) of 2-Y.

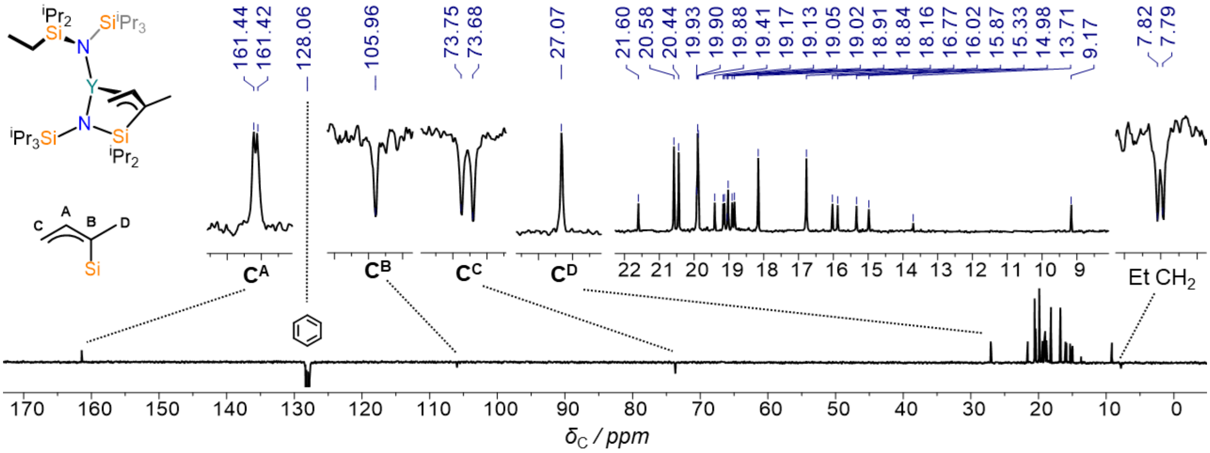

Fig. S5 | ^{13}C{^{1}H} DEPTQ NMR spectrum (125.79 MHz, C_6D_6, 298 K) of 2-Y.

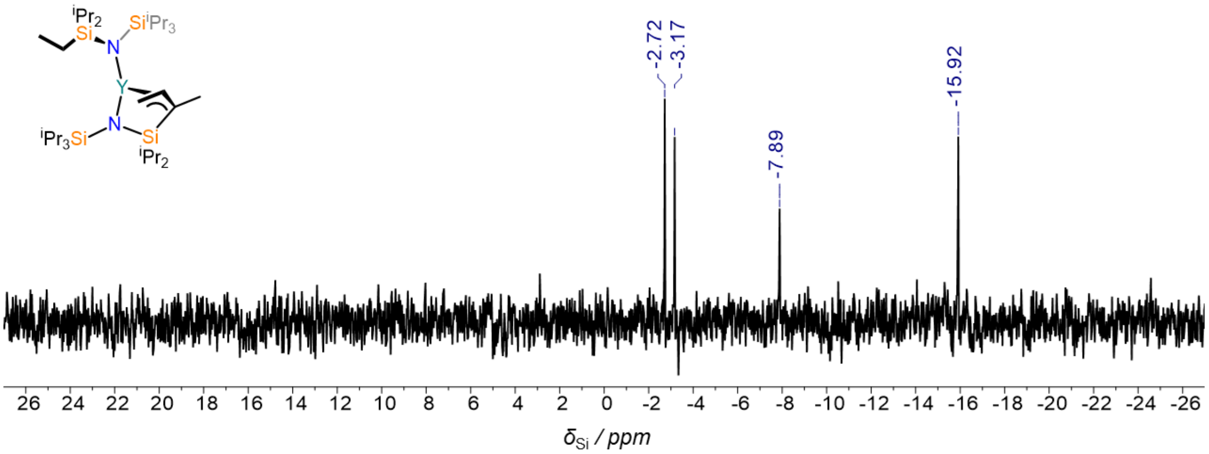

Fig. S6 | ^{29}Si{^{1}H} DEPT90 NMR spectrum (79.48 MHz, C_6H_5F, 298 K) of 2-Y.

3. Powder X-ray diffraction

Table S1 | Powder XRD cell parameters of 1-Dy, 1-Y and 5%Dy@1-Y at 100 K

	**1-Dy**	**1-Y**	**5%Dy@1-Y**
a, Å	15.9149(4)	15.9347(4)	15.9417(4)
b, Å	20.2535(4)	20.2254(6)	20.2284(6)
c, Å	22.8034(5)	22.7548(7)	22.7591(8)
α, °	90	90	90
β, °	92.5271(13)	92.476(2)	92.395(3)
γ, °	90	90	90

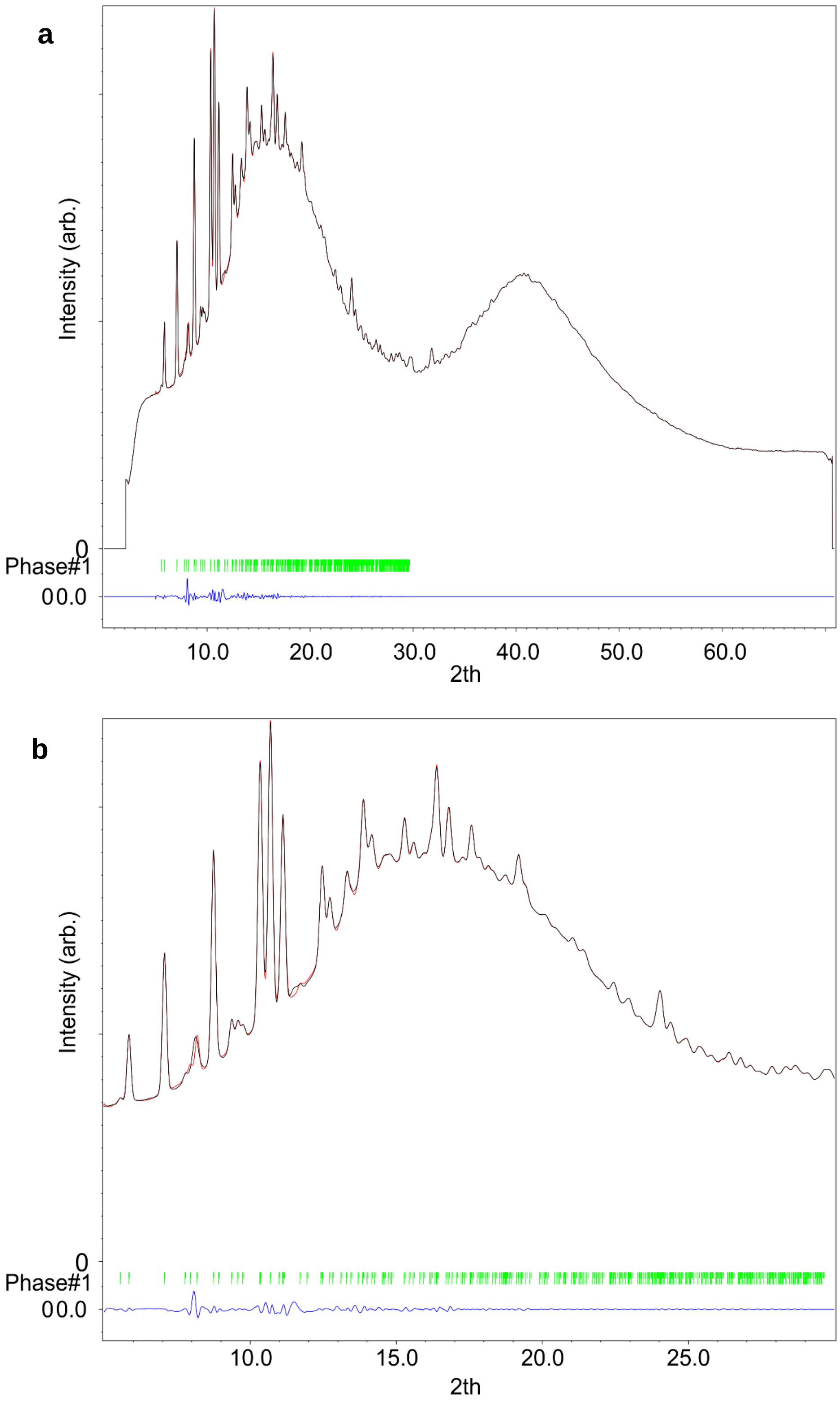

Fig. S7 | Powder XRD patterns of 1-Y. a, Full pattern. **b**, Selected range for Le Bail profile fitting. Black = observed data, red = calculated profile, green = reflection positions, blue = observed-calculated difference.

4. IR spectra

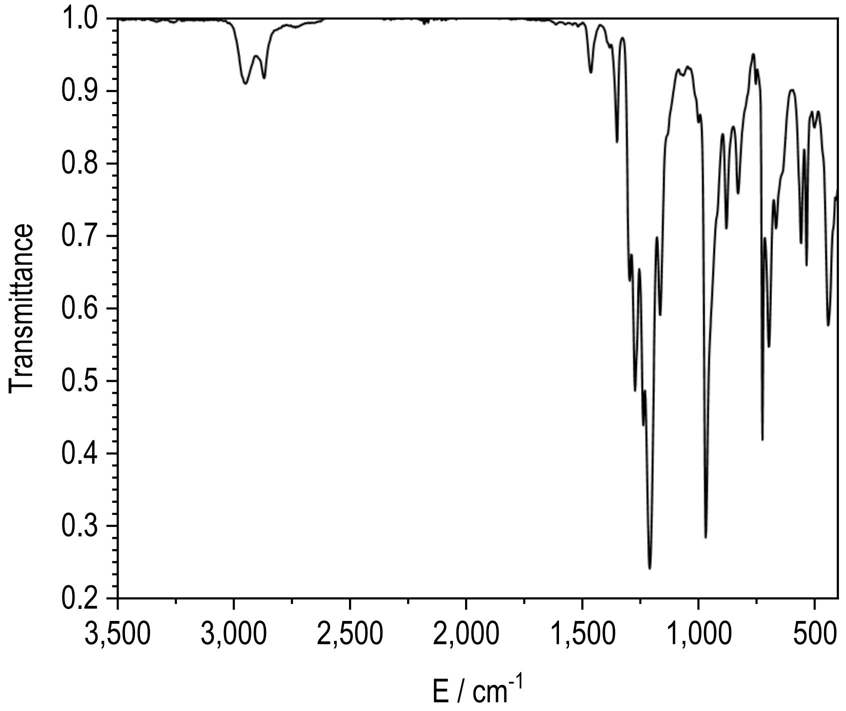

Fig. S8 | FT-IR (ATR) spectrum of microcrystalline 1-Y.

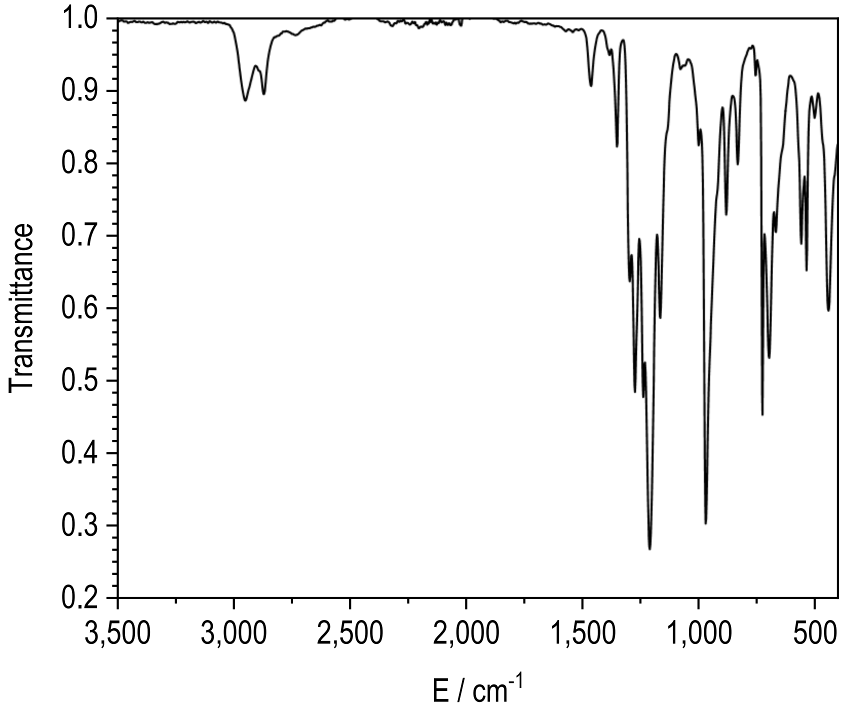

Fig. S9 | FT-IR (ATR) spectrum of microcrystalline 1-Dy.

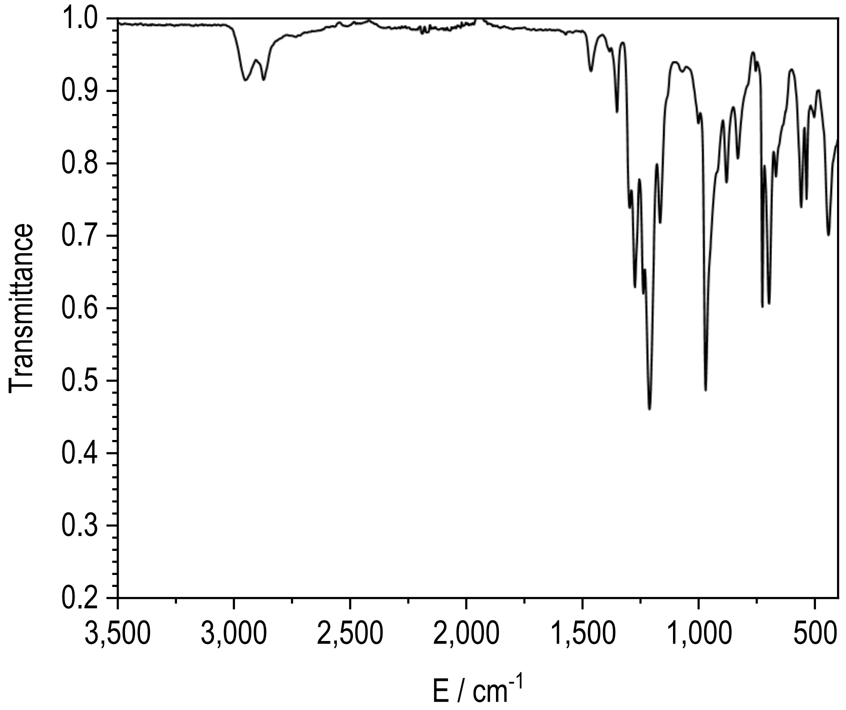

Fig. S10 | FT-IR (ATR) spectrum of microcrystalline 5%Dy@1-Y.

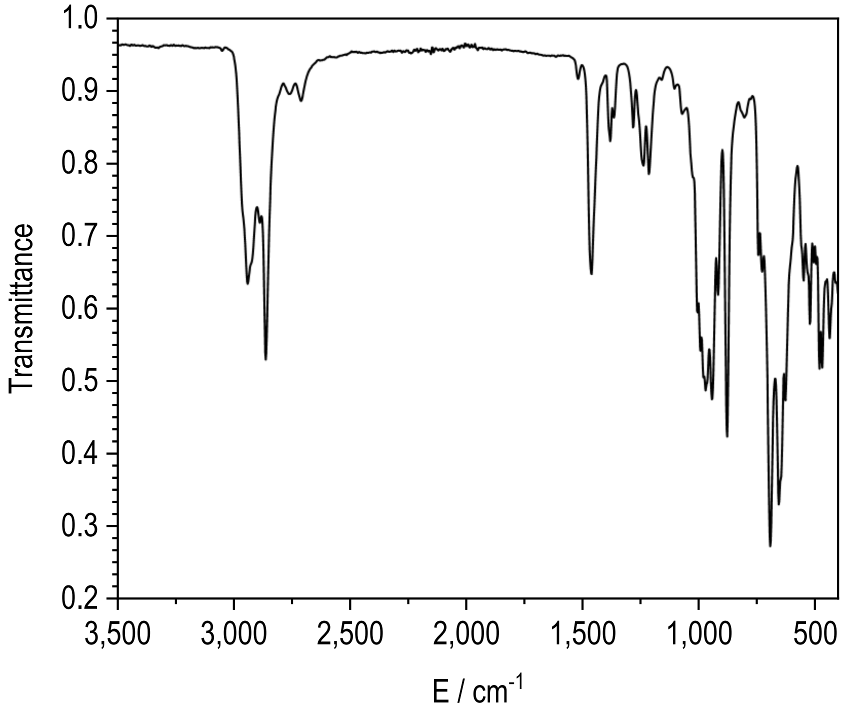

Fig. S11 | FT-IR (ATR) spectrum of microcrystalline 2-Y.

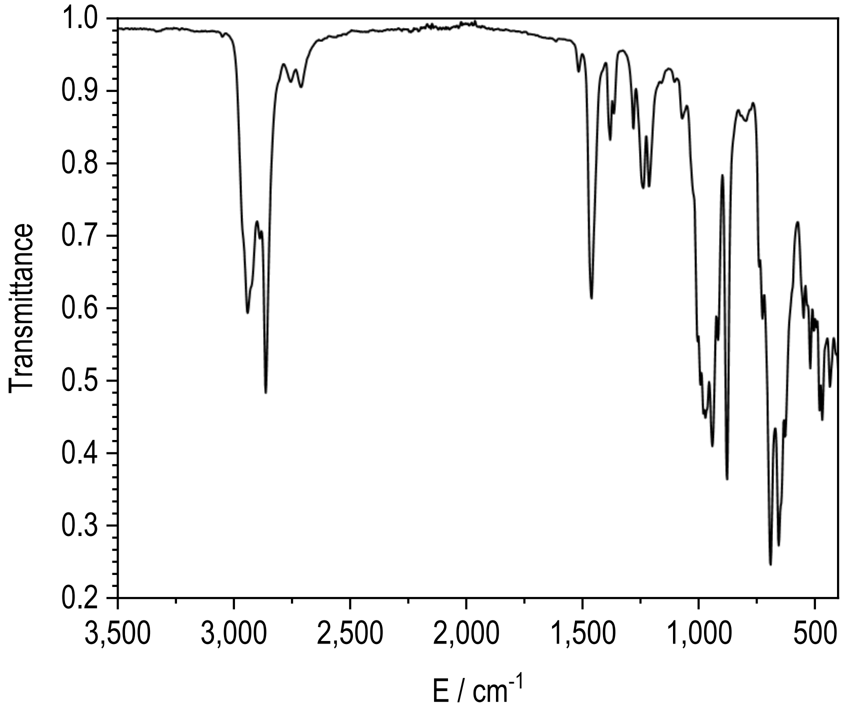

Fig. S12 | FT-IR (ATR) spectrum of microcrystalline 2-Dy.

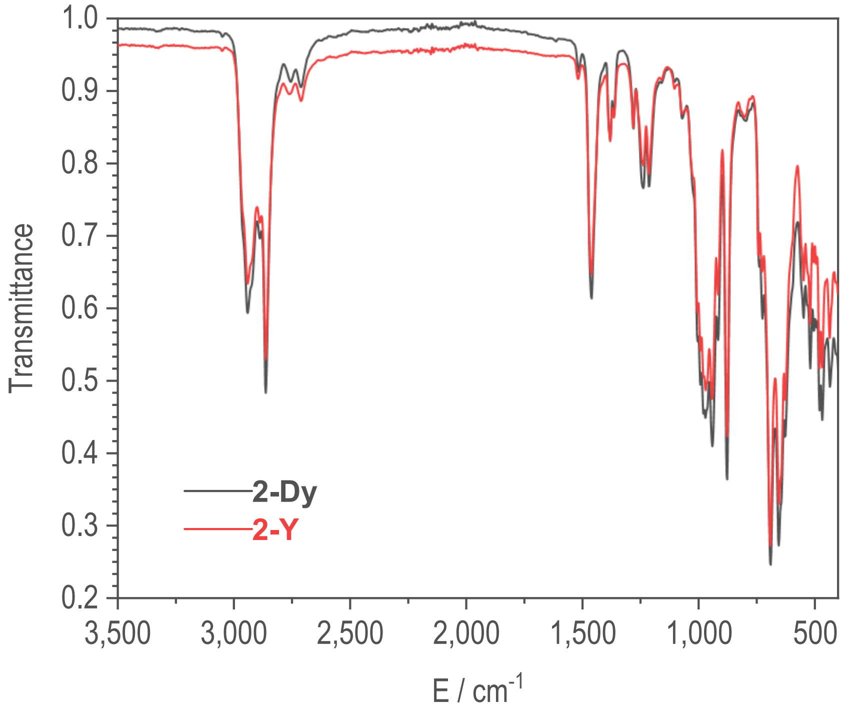

Fig. S13 | Overlaid FT-IR (ATR) spectra of microcrystalline 2-Ln.

5. Single crystal X-ray diffraction

The $\{OC(CF_3)_3\}$ substituents of the $[Al\{OC(CF_3)_3\}_4]^-$ WCAs are highly disordered in the datasets for **1-Y**, **1-Dy** and **5%Dy@1-Y**, which strongly influences the statistics for the fit of the models (i.e. leads to larger R_1 and wR_2), especially to the weaker higher angle data. This issue is in addition to the disorder of the silylamide-containing components, which occur due to the intrinsic flexibility of the metal coordination spheres, giving a wide range of metrical parameters. The disorder in these systems cannot be fully modelled while retaining a suitable data to parameter ratio for the given resolution of the datasets, making the overall fit to the data relatively poor compared to systems without such disorder. This is a known problem for crystal systems containing $[Al\{OC(CF_3)_3\}_4]^-$, as evidenced by papers cited in the manuscript (refs#12, 17 and 24). These previously reported datasets generally have high R_1 and wR_2 values, even when the data are modelled as effectively as possible and low goodness of fit metrics are obtained. Plots of $2F_o$-F_c show good agreement of the model of the main molecule with the electron density map, with the majority of deficiencies around the WCAs, confirmed by plots of F_o-F_c difference maps. The noise around the heavy metal sites in the F_o-F_c difference maps is common when collecting data for compounds containing heavy metals using CuKα radiation; the small size of the crystals precluded the use of a lower brilliance, shorter wavelength, MoKα source when collecting the data. This also limits the maximum resolution of the dataset that can be obtained, limiting the extent that disorder of the WCAs can be modelled to improve the fit.

Table S2 | Single-crystal diffraction data for 1-Dy, 1-Y, 5%Dy@1-Y, 2-Dy and 2-Y

Parameter	1-Dy	1-Y	5%Dy@1-Y	2-Dy	2-Y
Formula	$C_{52}H_{82}AlDyF_{36}N_2O_4Si_4$	$C_{52}H_{82}AlF_{36}N_2O_4Si_4Y$	$C_{52}H_{82}AlDy_{0.07}F_{36}N_2O_4Si_4Y_{0.93}$	$C_{36}H_{81}DyN_2Si_4$	$C_{36}H_{81}N_2Si_4Y$
Fw	1785.03	1711.44	1715.12	816.88	743.29
Cryst size, mm	0.142×0.098×0.046	0.141×0.089×0.073	0.100×0.072×0.044	0.122×0.069×0.055	0.136×0.098×0.039
Cryst system	Monoclinic	Monoclinic	Monoclinic	Triclinic	Triclinic
Space group	*P*21/*c*	*P*21/*c*	*P*21/*c*	*P*–1	*P*–1
Temperature, K	99.96(18)	99.99(10)	99.96(14)	120.00(10)	100.02(10)
a, Å	15.88836(8)	15.9105(3)	15.9040(7)	10.4509(3)	10.4766(3)
b, Å	20.26883(11)	20.2210(3)	20.2350(11)	11.6812(4)	11.6346(4)
c, Å	22.81604(12)	22.7413(3)	22.7636(12)	19.8755(6)	19.9866(5)
α, °	90	90	90	102.137(3)	102.167(2)
β, °	92.5864(5)	92.4904(14)	92.523(4)	91.832(3)	91.694(2)
γ, °	90	90	90	115.211(3)	115.506(3)
V, $Å^3$	7340.16(7)	7309.6(2)	7318.6(6)	2125.83(13)	2129.29(12)
Z	4	4	4	2	2
ρ_{calcd}, g cm^{-3}	1.615	1.555	1.557	1.276	1.159
μ, mm^{-1}	7.412	3.098	3.311	10.651	3.176
No. reflections made	88625	50029	43247	22387	25958
No. unique reflns, R_{int}	15157, 0.0346	14900, 0.0401	14929, 0.0571	8506, 0.0317	8458, 0.0369
No. reflns $F^2 > 2\sigma(F^2)$	14014	11093	9137	8287	7847
Transmn coeff range	0.387-1.000	0.867-1.000	0.896-1.000	0.341-0.608	0.908-1.000
R, R_w[a] ($F^2 > 2\sigma(F^2)$)	0.0664, 0.1935	0.0903, 0.2683	0.0938, 0.2621	0.0718, 0.1788	0.1070, 0.2536
R, R_w[a] (all data)	0.0693, 0.1974	0.1090, 0.2919	0.1328, 0.3036	0.0729, 0.1792	0.1112, 0.2556
S[a]	1.065	1.261	1.085	1.190	1.199
Params, Restraints	1795, 6365	1794, 6401	1795, 6429	534, 1176	534,1170
Max/min diffmap, $eÅ^{-3}$	1.526, –0.981	1.066, –0.548	1.409, –0.698	0.806, –1.934	0.948, -1.525

[a] Conventional $R = \Sigma||F_o| - |F_c||/\Sigma|F_o|$; $R_w = [\Sigma w(F_o^2 - F_c^2)^2/\Sigma w(F_o^2)^2]^{1/2}$; $S = [\Sigma w(F_o^2 - F_c^2)^2/\text{no. data} - \text{no. params})]^{1/2}$ for all data.

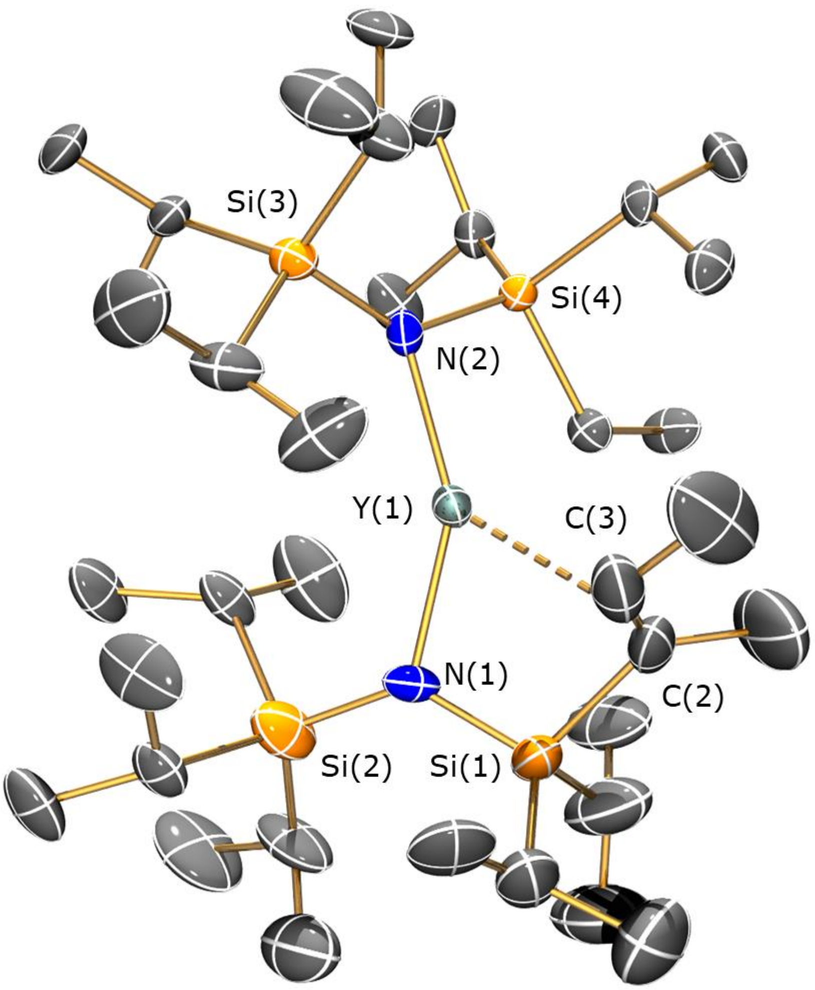

Fig. S14 | Molecular structure of the major component of the cation of 1-Y at 99.99(10) K. Displacement ellipsoids set at 30% probability level. Hydrogen atoms and counter-anion omitted for clarity. The Y atom is teal, N atoms are blue, Si atoms are orange and C atoms are grey. Selected distances (Å) and angles (°): Major component (0.566(6)), **1-Y-A**: Y1–N1, 2.197(9); Y1–N2, 2.199(9); Y1···C2, 2.83(2); Y1···C3, 2.75(2); C2–C3, 1.32(2); N1–Y1–N2, 147.7(6). Minor component (0.434(6)), **1-Y-B**: Y1–N1, 2.186(12); Y1–N2, 2.200(9); Y1···C2, 2.707(16); Y1···C3, 2.92(2); C2–C3, 1.351(15); N1–Y1–N2, 157.5(7).

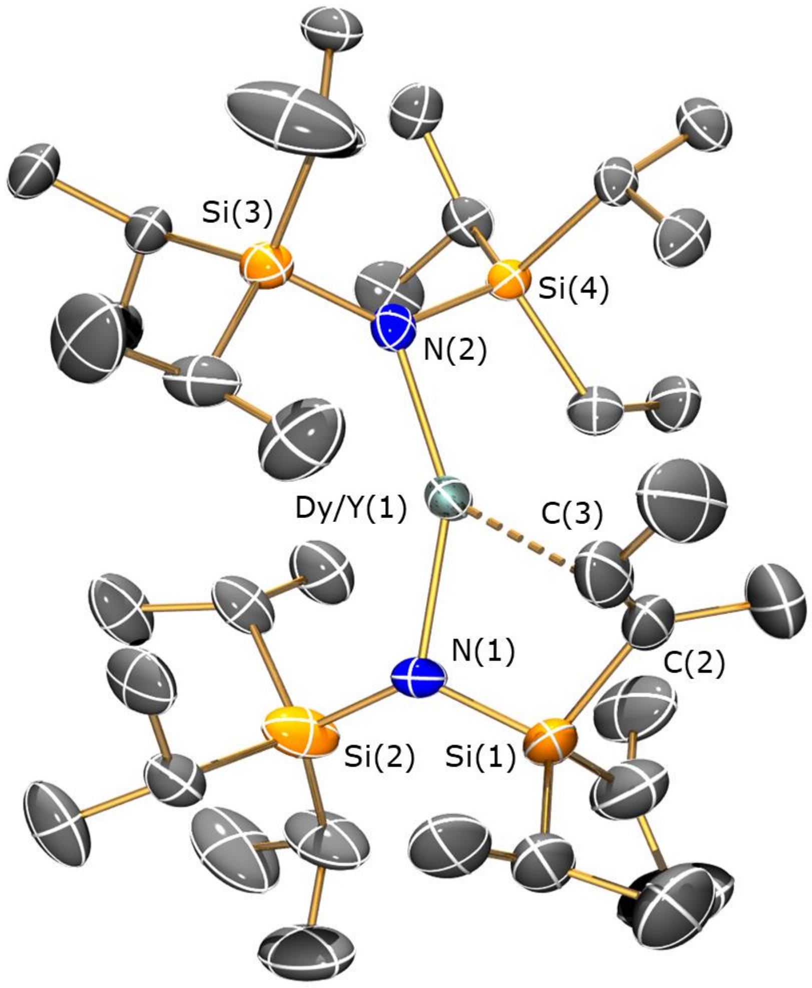

Fig. S15 | Molecular structure of the major component of the cation of 5%Dy@1-Y at 99.96(14) K. Displacement ellipsoids set at 30% probability level. Hydrogen atoms and counter-anion omitted for clarity. The Dy/Y atom is teal, N atoms are blue, Si atoms are orange and C atoms are grey. Bond distances and angles of the two components used to model disorder are not listed as these are not reliable, but the connectivity is clear-cut.

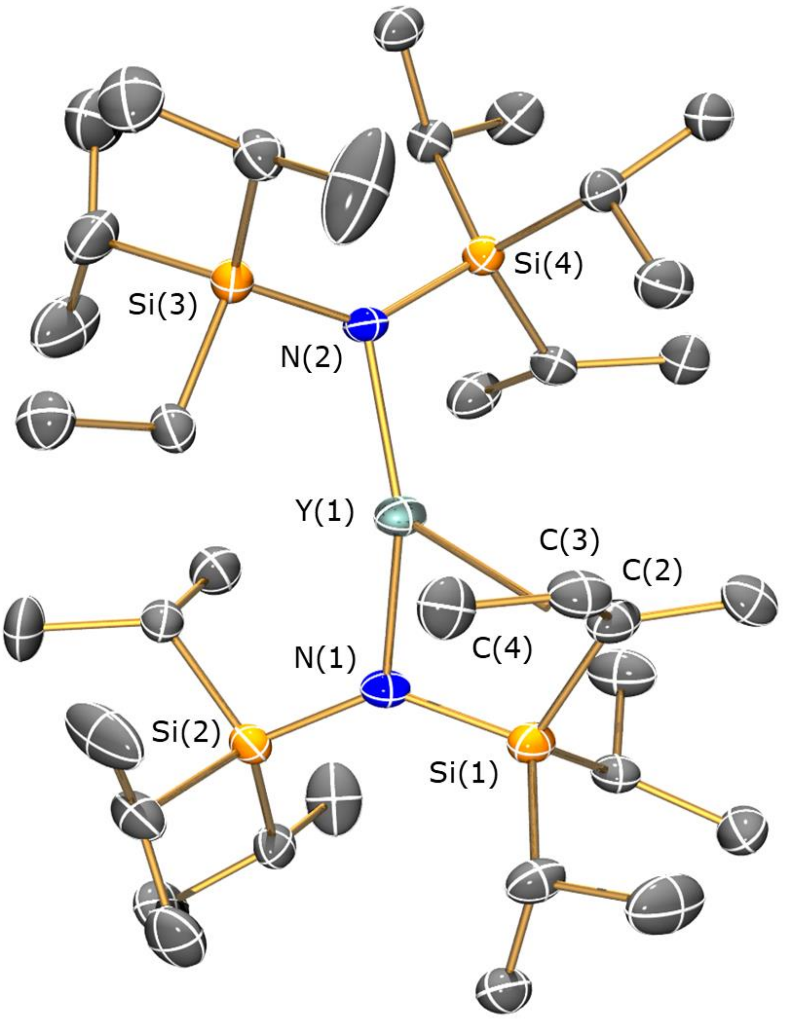

Fig. S16 | Molecular structure of the major component of 2-Y at 100.02(10) K. Displacement ellipsoids set at 50% probability level. Hydrogen atoms omitted for clarity. The Dy atom is teal, N atoms are blue, Si atoms are orange and C atoms are grey. Selected distances (Å) and angles (°): Major component (0.588(2)), **2-Y-A**: Y1–N1, 2.253(6); Y1–N2, 2.274(5); Y1···C2, 2.56(3) Å; Y1···C3, 2.63(4); Y1···C4, 2.499(14); C2–C3, 1.41(2); C3–C4, 1.46(3); N1–Y1–N2, 143.1(2). Minor component (0.412(2)), **2-Y-B**: Y1A–N1, 2.265(6); Y1A–N2, 2.279(6); Y1A···C2A, 2.51(5); Y1A···C3A, 2.60(5); Y1A···C4A, 2.49(2); C2A–C3A, 1.40(2); C3A–C4A, 1.46(3); N1–Y1A–N2, 141.9(2).

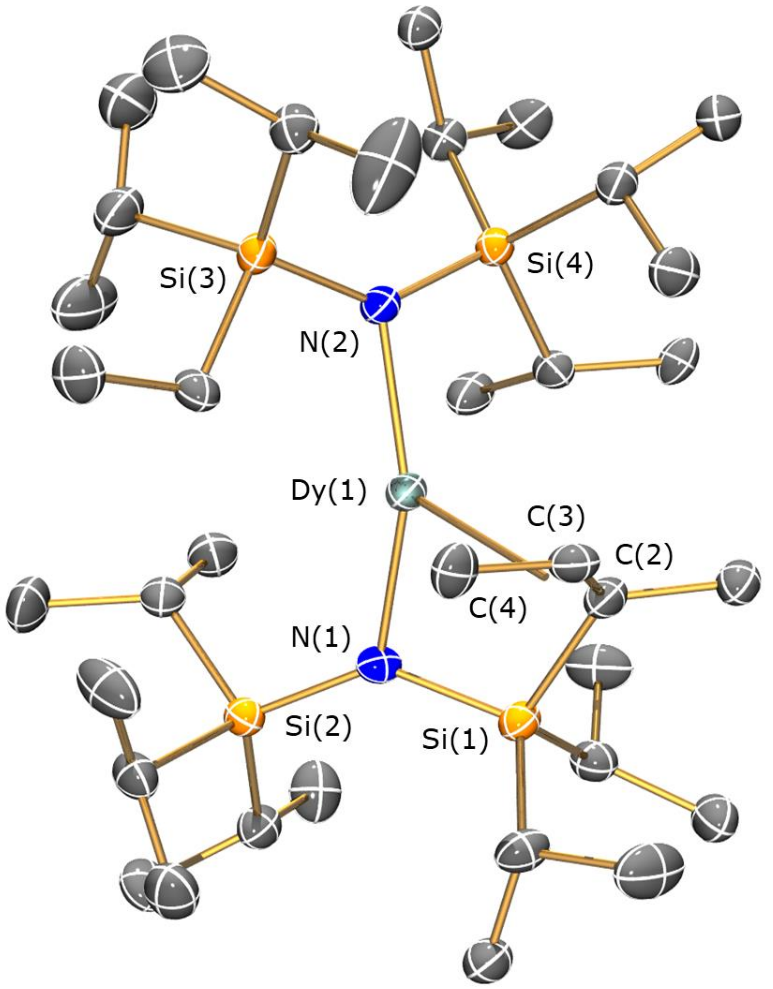

Fig. S17 | Molecular structure of the major component of 2-Dy at 120.00(10) K. Displacement ellipsoids set at 50% probability level. Hydrogen atoms omitted for clarity. The Dy atom is teal, N atoms are blue, Si atoms are orange and C atoms are grey. Selected distances (Å) and angles (°): Major component (0.7322(15)), **2-Dy-A**: Dy1–N1, 2.248(5); Dy1–N2, 2.290(5); Dy1···C2, 2.52(2); Dy1···C3, 2.57(2); Dy1···C4, 2.511(10); C2–C3, 1.422(15); C3–C4, 1.406(19); N1–Dy1–N2, 144.8(2). Minor component (0.2678(15)), **2-Dy-B**: Dy1A–N1, 2.2567(5); Dy1A–N2, 2.285(5); Dy1A···C2A, 2.67(6); Dy1A···C3A, 2.64(6); Dy1A···C4A, 2.50(3); C2A–C3A, 1.42(2); C3A–C4A, 1.41(3); N1–Dy1A–N2, 144.4(2).

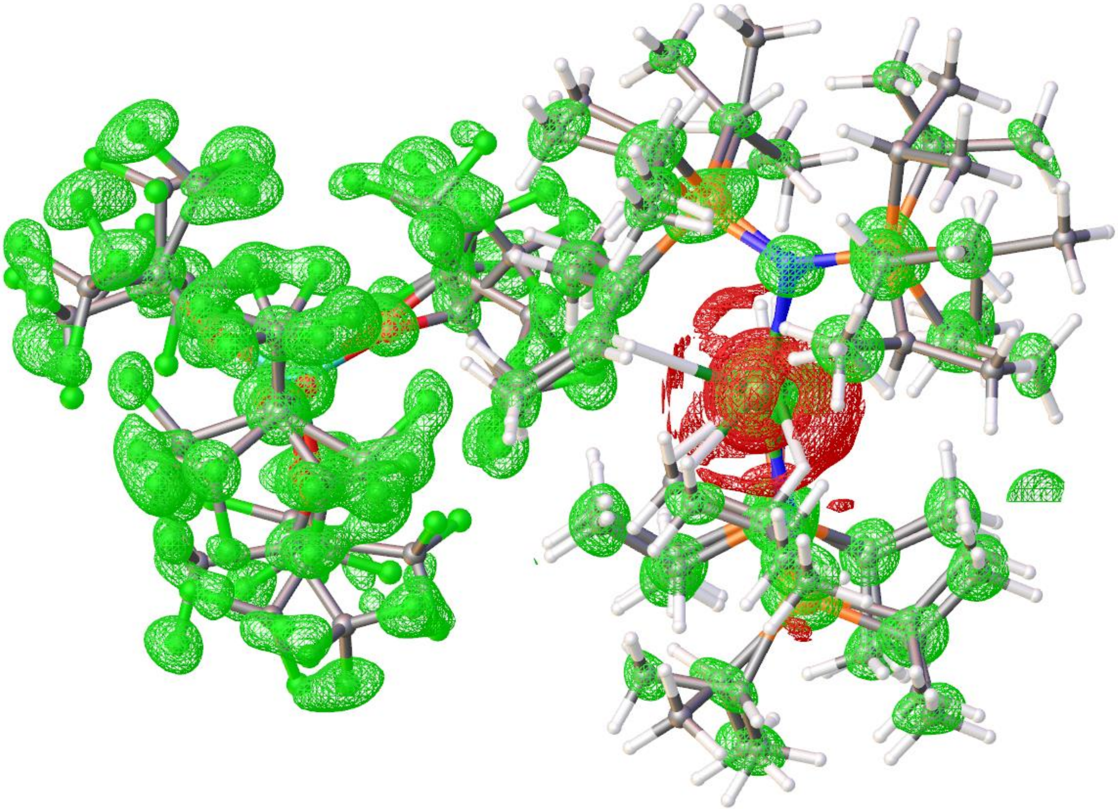

Fig. S18 | Electron density plot of $2F_o–F_c$ for 1-Dy @ 2 $e^{–}/Å^3$.

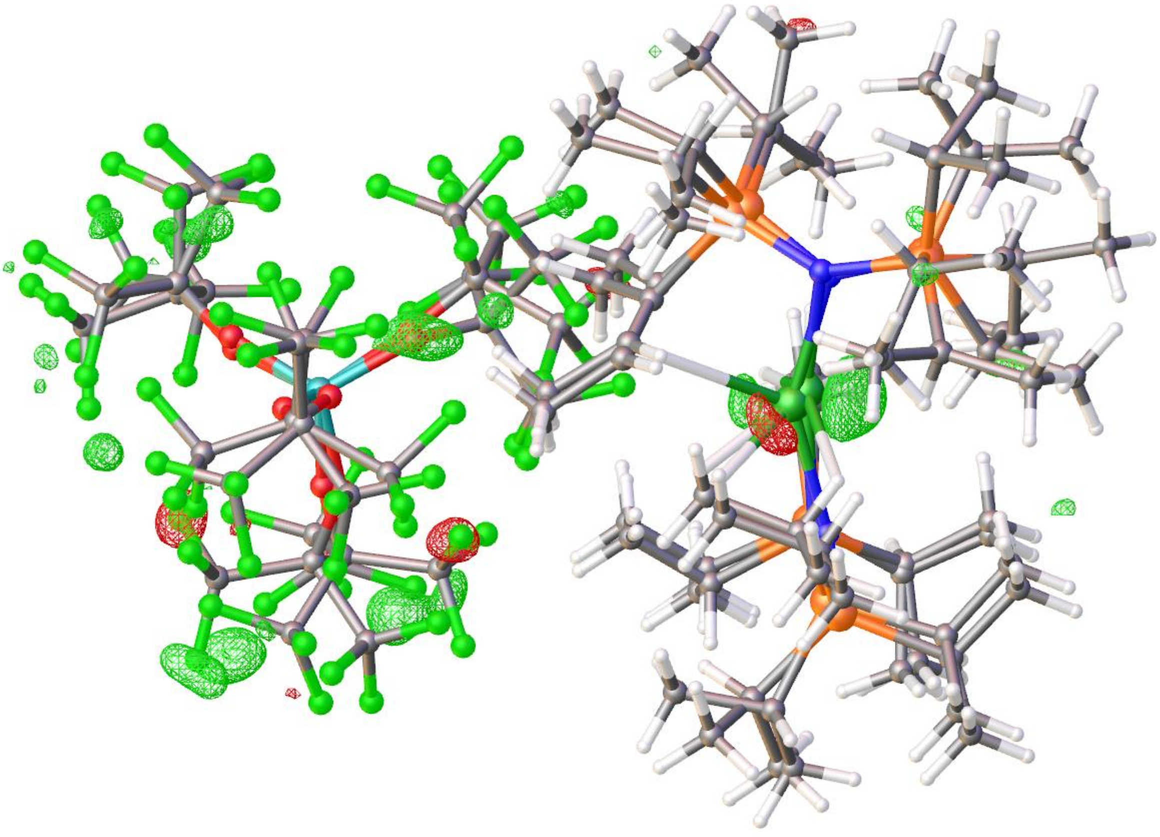

Fig. S19 | Electron density plot of $F_o–F_c$ for 1-Dy @ 0.5 $e^{–}/Å^3$.

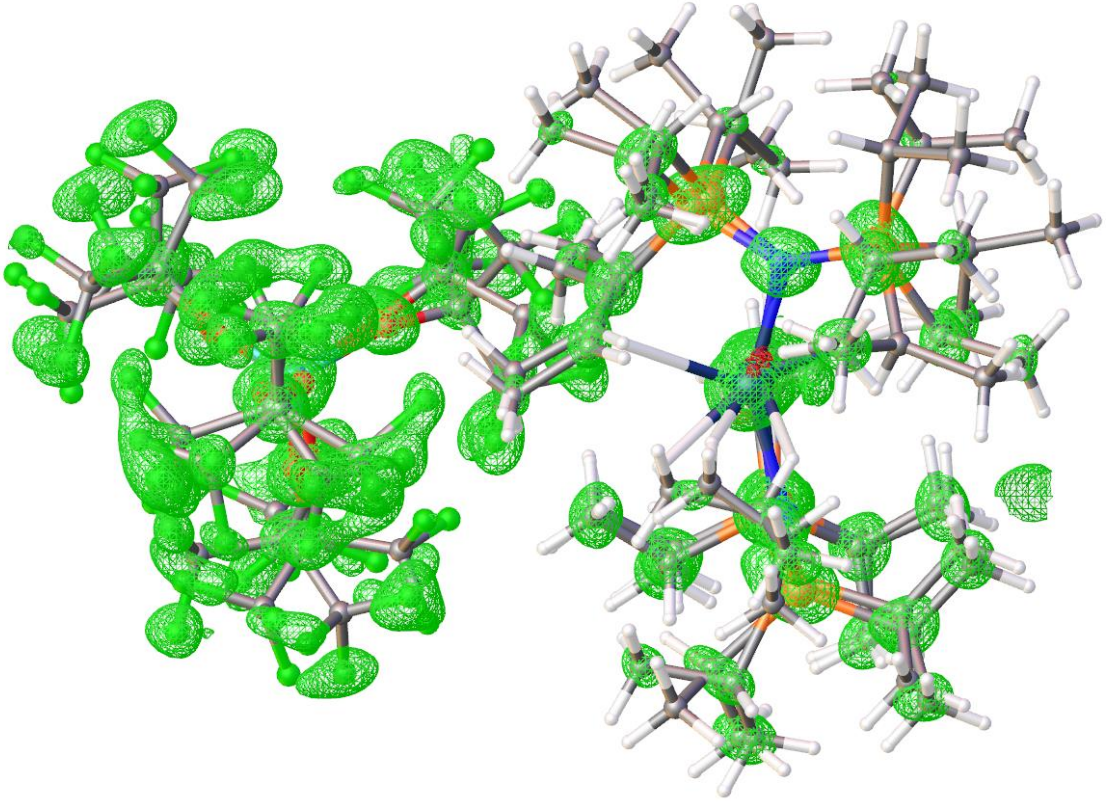

Fig. S20 | Electron density plot of $2F_o–F_c$ for 1-Y @ 2 $e^–/Å^3$.

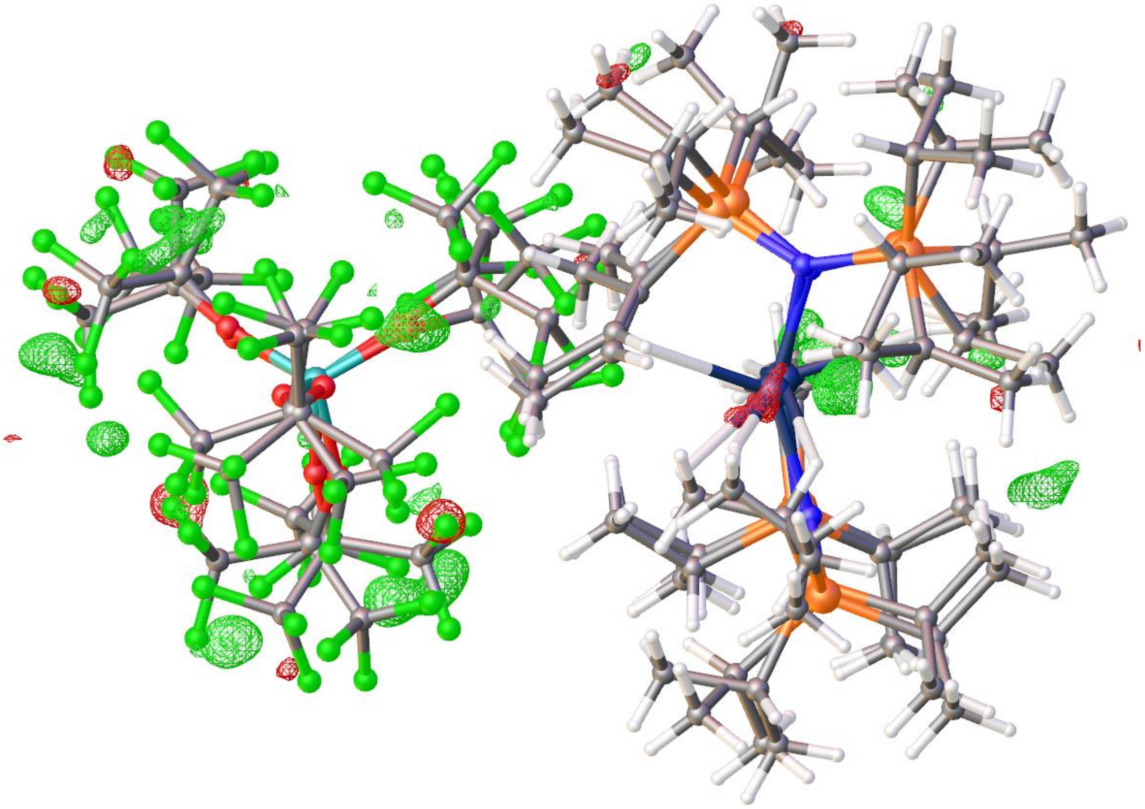

Fig. S21 | Electron density plot of $F_o–F_c$ for 1-Y @ 0.5 $e^–/Å^3$.

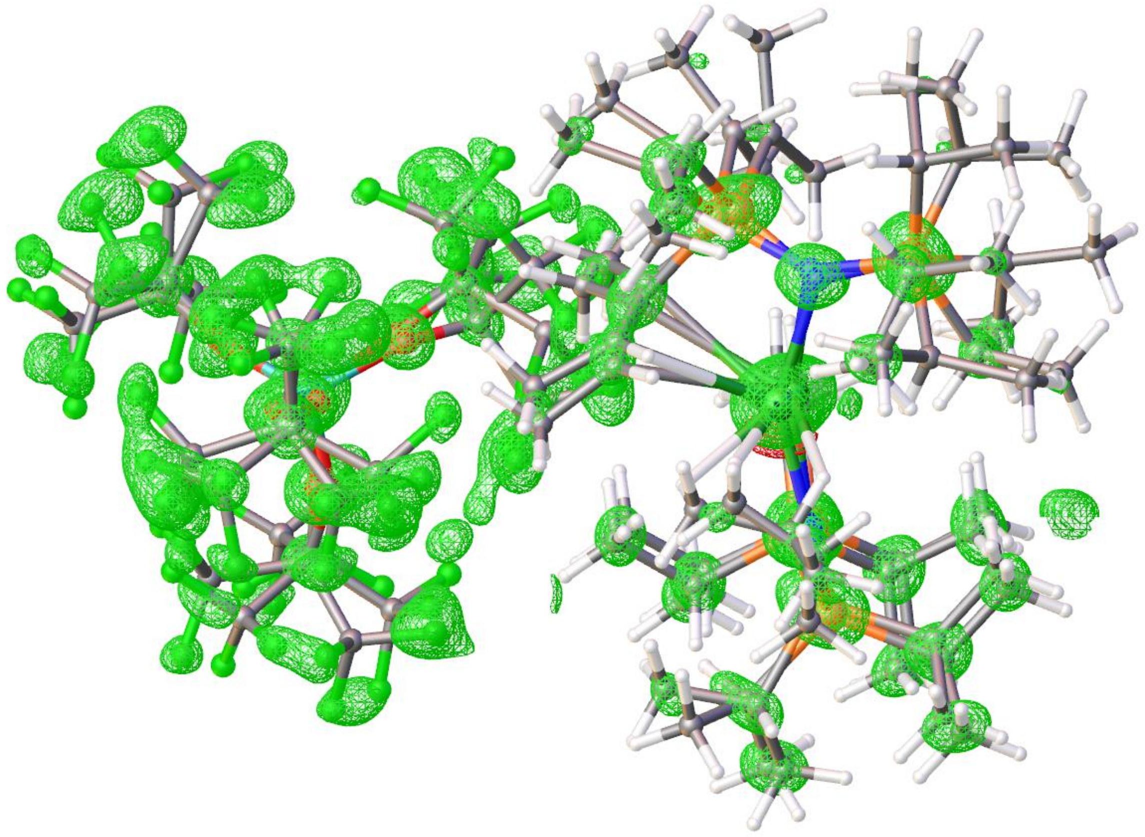

Fig. S22 | Electron density plot of $2F_o–F_c$ for 5%Dy@1-Y @ 2 $e^–/Å^3$.

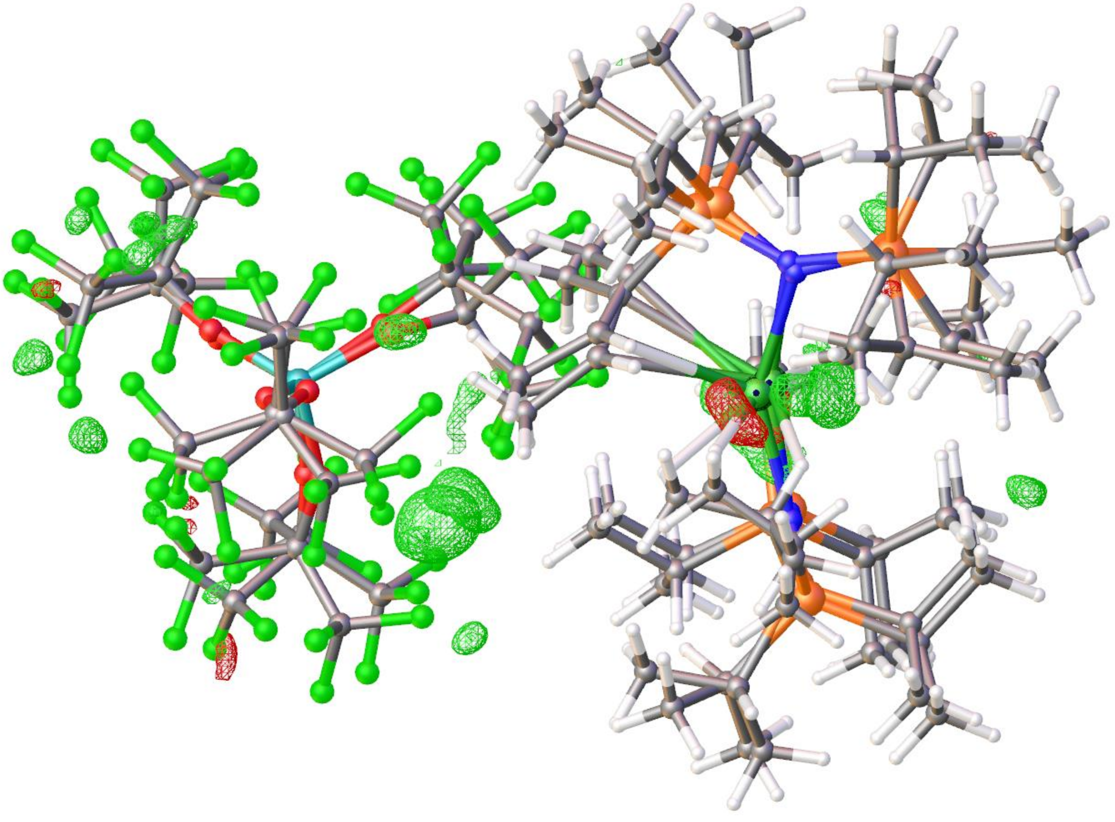

Fig. S23 | Electron density plot of $F_o–F_c$ for 5%Dy@1-Y @ 0.5 $e^–/Å^3$.

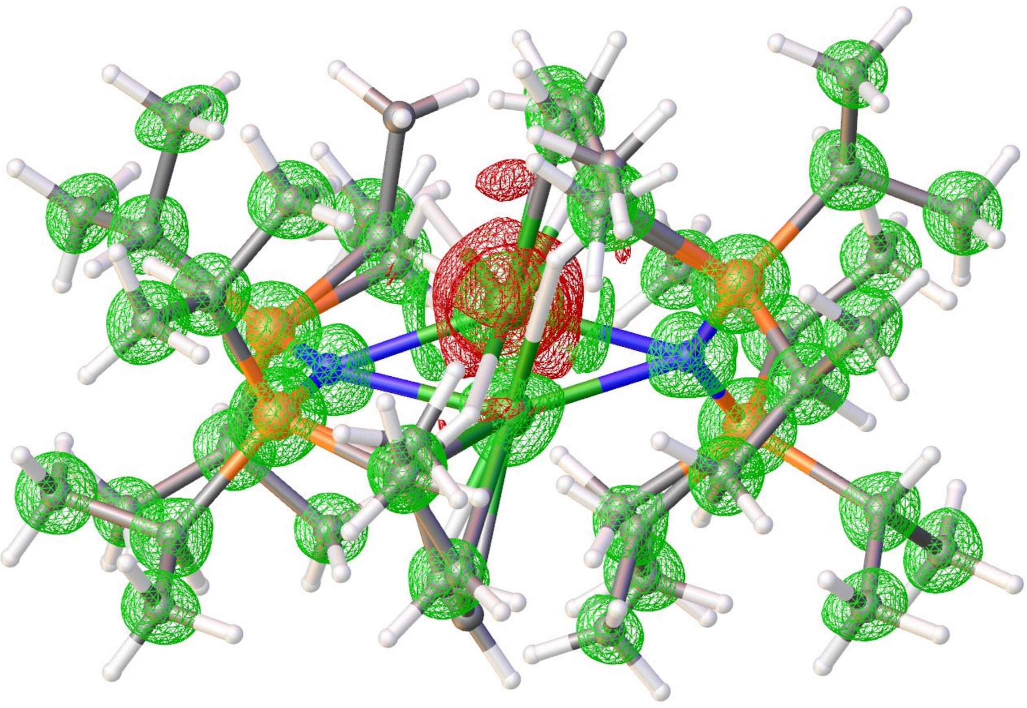

Fig. S24 | Electron density plot of 2F_o–F_c for 2-Dy @ 2.6 $e^-/Å^3$.

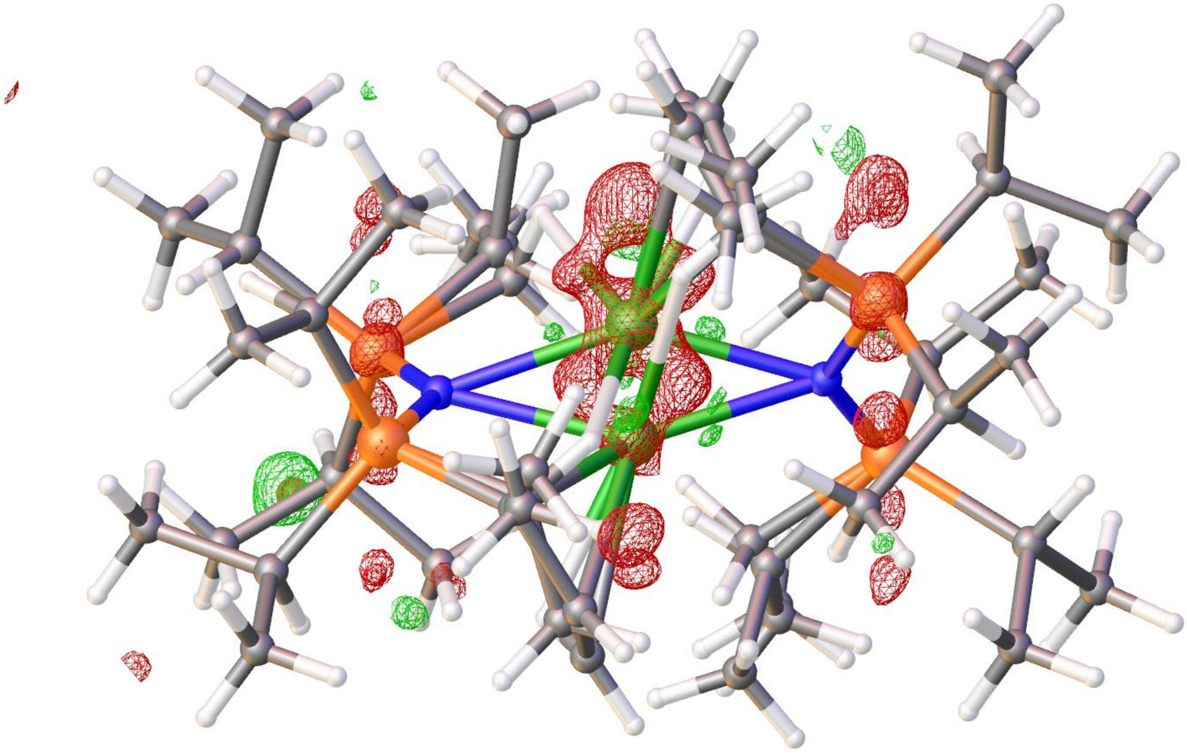

Fig. S25 | Electron density plot of F_o–F_c for 2-Dy @ 0.5 $e^-/Å^3$.

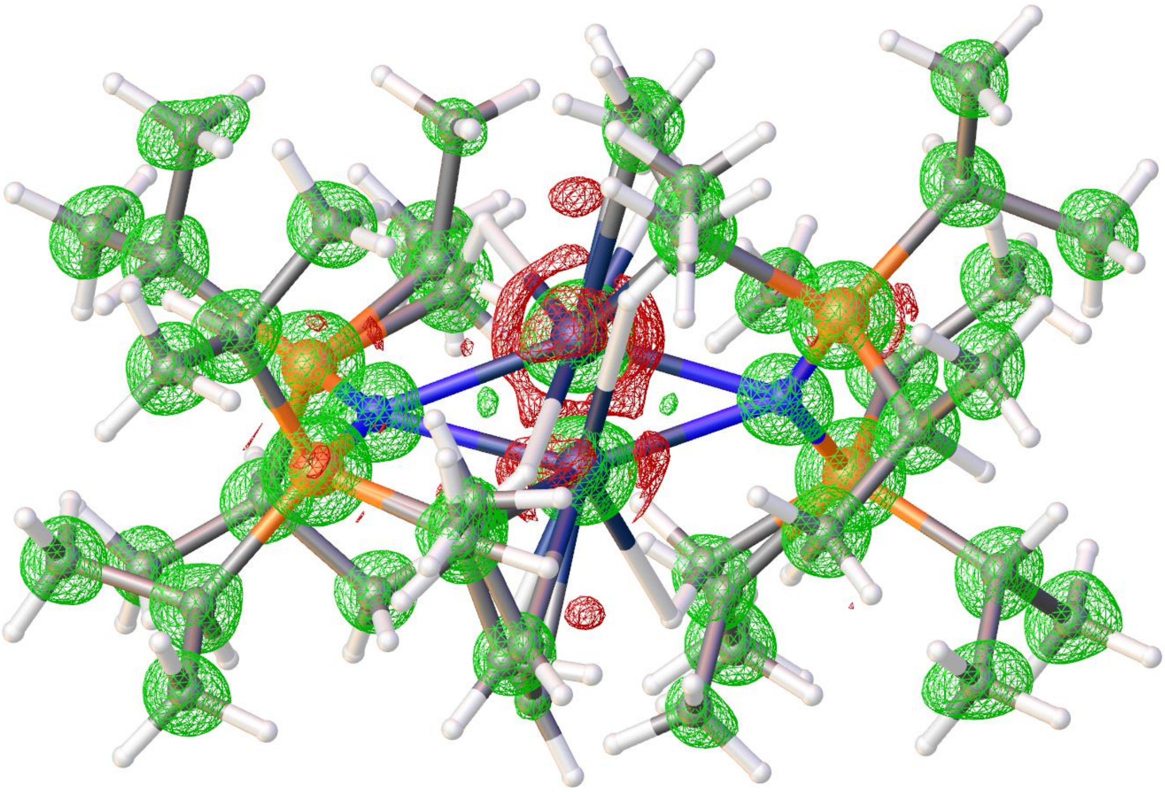

Fig. S26 | Electron density plot of $2F_o–F_c$ for 2-Y @ 2.6 $e^-/Å^3$.

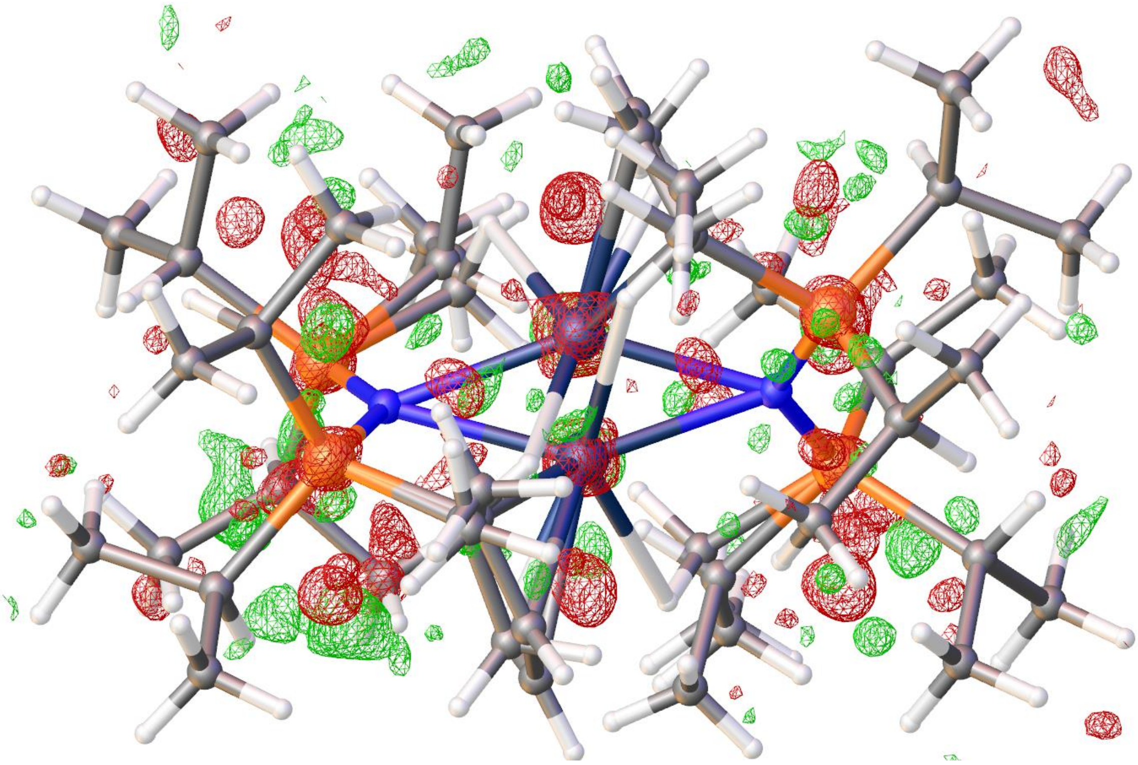

Fig. S27 | Electron density plot of $F_o–F_c$ for 2-Y @ 0.5 $e^-/Å^3$.

6. DFT calculations

Table S3 | Bond critical point properties of 1-Y at the DFT optimised geometry[a]

**Bond critical point**	***ρ* / a.u.**	**∇^2 / a.u.**	***G* / a.u.**
Y–N(amide)	0.0869	0.2720	0.0872
Y–N(amide’)	0.0842	0.2675	0.0847
Y–C(alkene)	0.0336	0.0775	0.0227

[a] At the bond critical point, ρ = electron density; ∇^2 = Laplacian of the electron density, G = kinetic energy density.

7. CASSCF electronic structure

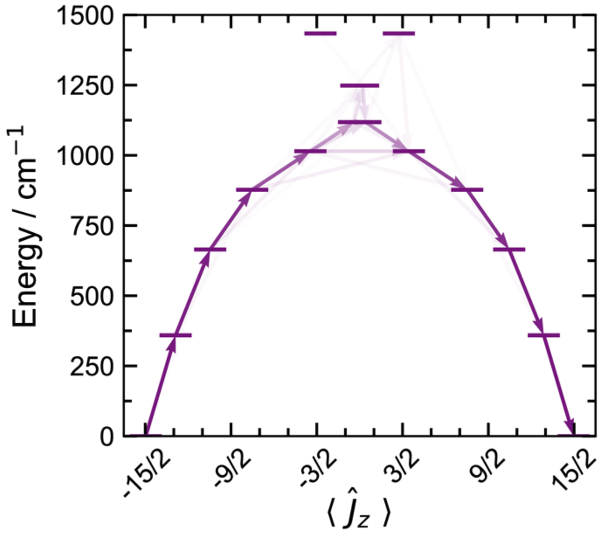

Fig. S28 | Energy barrier to magnetisation reversal for a model of 2-Dy from CASSCF-SO calculations. Arrows represent the Orbach magnetisation reversal pathway, where the opacity of the arrows is proportional to the transition probability approximated with the average matrix elements of magnetic moment connecting the states, $\gamma_{ij} = (1/3)[|\langle i|\mu_x|j\rangle|^2 + |\langle i|\mu_y|j\rangle|^2 + |\langle i|\mu_z|j\rangle|^2]$, normalized from each departing state and commencing from |–15/2>.

Table S4 | CASSCF-SO-calculated electronic structure of 2-Dy using the crystal structure geometry of 2-Dy in zero-field[a]

<table>
<tr><th>Energy (cm^{-1})</th><th>Energy (K)</th><th>g_x</th><th>g_y</th><th>g_z</th><th>Angle[b] (deg)</th><th><J_z></th><th>Wavefunction</th></tr>
<tr><td>0.00</td><td>0.00</td><td>0.00</td><td>0.00</td><td>19.87</td><td>0.00</td><td>±7.48</td><td>94.73% |±15/2></td></tr>
<tr><td>359.23</td><td>513.26</td><td>0.01</td><td>0.01</td><td>16.99</td><td>4.19</td><td>±6.44</td><td>91.54% |±13/2></td></tr>
<tr><td>664.67</td><td>949.67</td><td>0.20</td><td>0.24</td><td>14.07</td><td>13.83</td><td>±5.23</td><td>79.47% |±11/2> + 8.34% |±7/2></td></tr>
<tr><td>877.37</td><td>1253.57</td><td>0.54</td><td>0.85</td><td>11.40</td><td>30.72</td><td>±3.76</td><td>48.14% |±9/2> + 21.28% |±5/2> + 8.80% |±11/2> + 6.54% |±7/2></td></tr>
<tr><td>1014.64</td><td>1449.70</td><td>3.75</td><td>6.30</td><td>7.80</td><td>62.35</td><td>±1.72</td><td>22.73% |±9/2> + 20.96% |±3/2> + 14.55% |±1/2> + 13.25% |±7/2> + 7.30% |∓3/2></td></tr>
<tr><td>1118.28</td><td>1597.78</td><td>1.78</td><td>3.87</td><td>12.19</td><td>86.98</td><td>±0.20</td><td>24.80% |±7/2> + 16.71% |±1/2> + 16.43% |∓5/2> + 9.57% |∓1/2> + 8.74% |∓3/2> + 6.86% |∓9/2> + 5.25% |∓7/2></td></tr>
<tr><td>1248.45</td><td>1783.77</td><td>0.07</td><td>0.14</td><td>16.70</td><td>88.91</td><td>±0.12</td><td>22.48% |±5/2> + 17.98% |∓7/2> + 14.28% |∓1/2> + 14.26% |±3/2> + 6.95% |±1/2> + 5.75% |∓3/2></td></tr>
<tr><td>1433.93</td><td>2048.78</td><td>0.03</td><td>0.07</td><td>19.32</td><td>79.15</td><td>±1.37</td><td>26.74% |±3/2> + 17.44% |∓1/2> + 15.33% |∓5/2> + 11.13% |±7/2> + 10.13% |±5/2> + 6.73% |±1/2></td></tr>
</table>

[a] Each row corresponds to a Kramers doublet. All data is directly from the CASSCF-SO calculation, except for the Wavefunction which is necessarily obtained by projection onto a model space of the $^6H_{15/2}$ term. [b] The angle between the g_z value of the excited Kramers doublet and the ground Kramers doublet.

8. Magnetic data

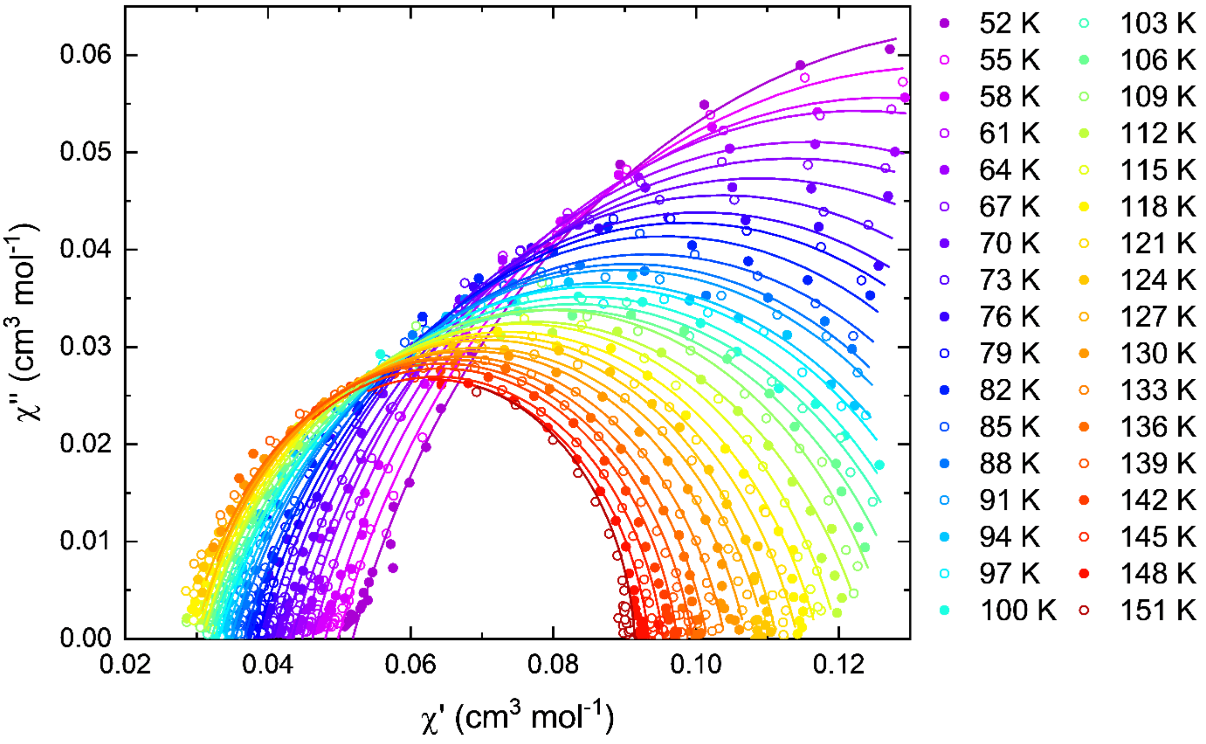

Fig. S29 | A.c. field susceptibility Cole-Cole plot for 1-Dy. Fitting of frequency-dependent out-of-phase (χ″) vs. in-phase (χ′) susceptibility in zero d.c. field to the generalised Debye model in CCFIT-2.

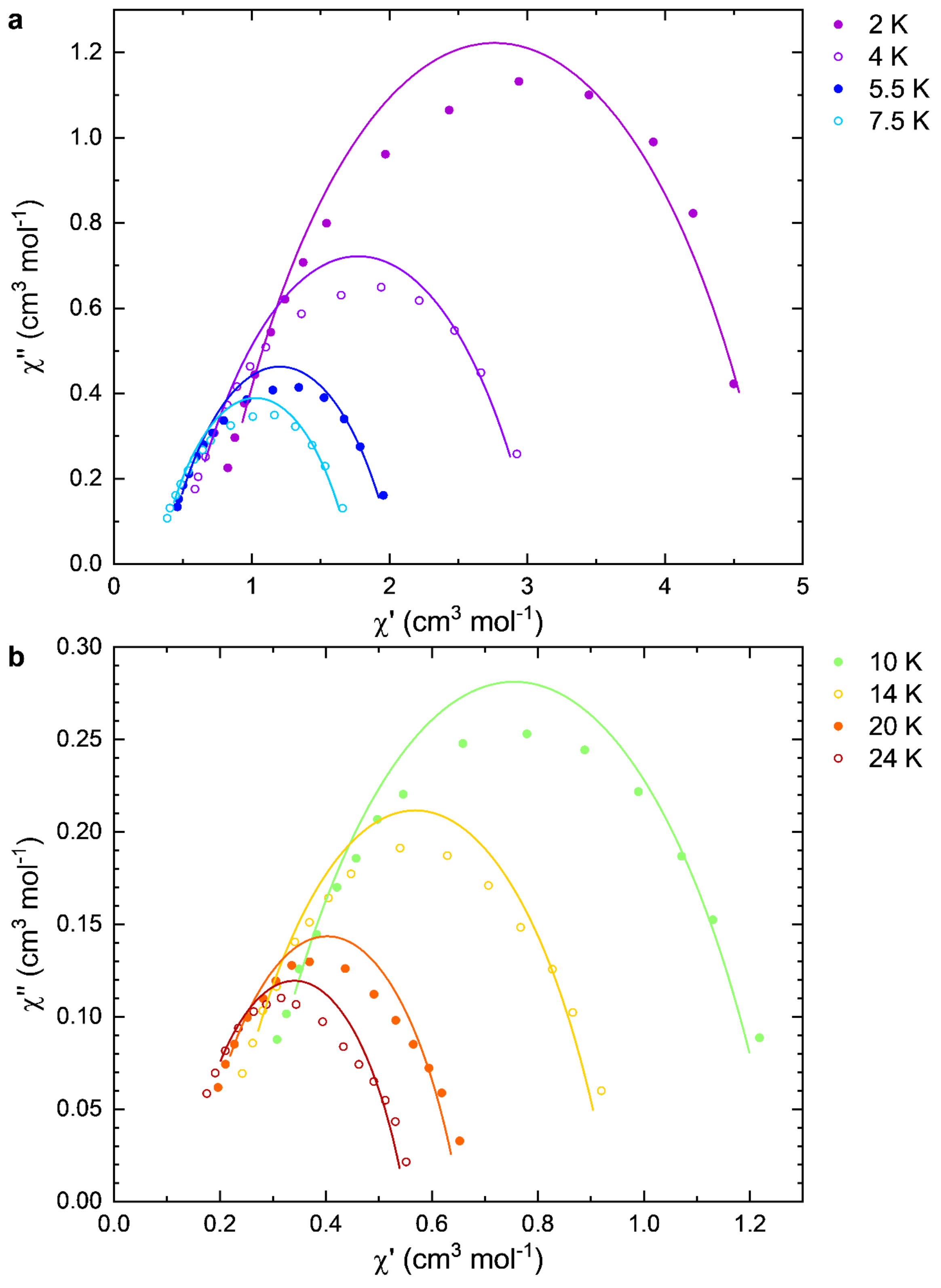

Fig. S30 | Waveform susceptibility Cole-Cole plot for 1-Dy. Fitting of frequency-dependent out-of-phase (χ″) vs. in-phase (χ′) susceptibility in zero d.c. field to the generalised Debye model in CCFIT-2. **a**, 2–7.5 K. **b**, 10–24 K.

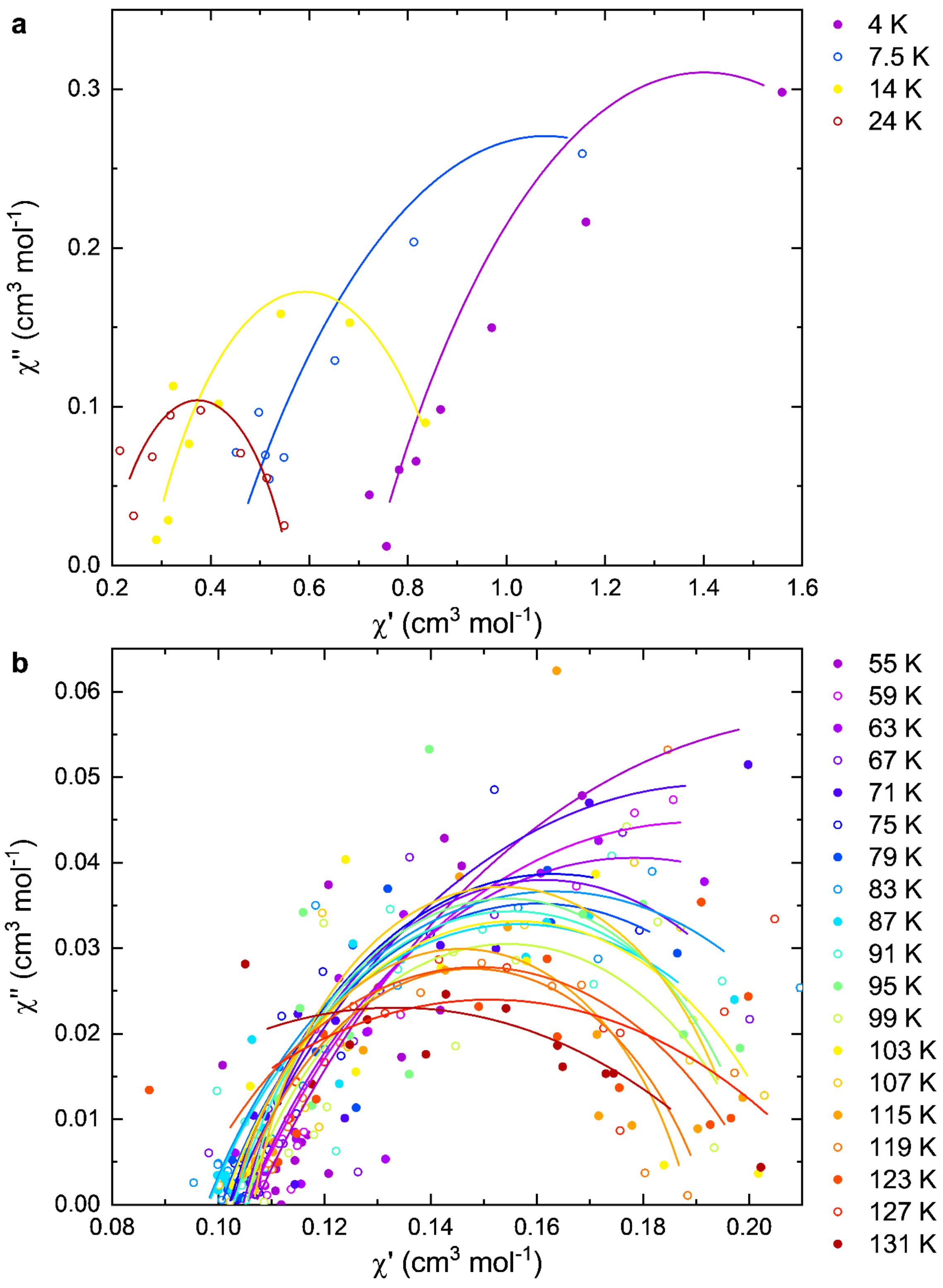

Fig. S31 | Cole-Cole plots for 5%Dy@1-Y. Fitting of frequency-dependent out-of-phase (χ″) vs. in-phase (χ′) susceptibility in zero d.c. field to the generalised Debye model in CCFIT-2. **a**, A.c. data. **b**, Waveform data.

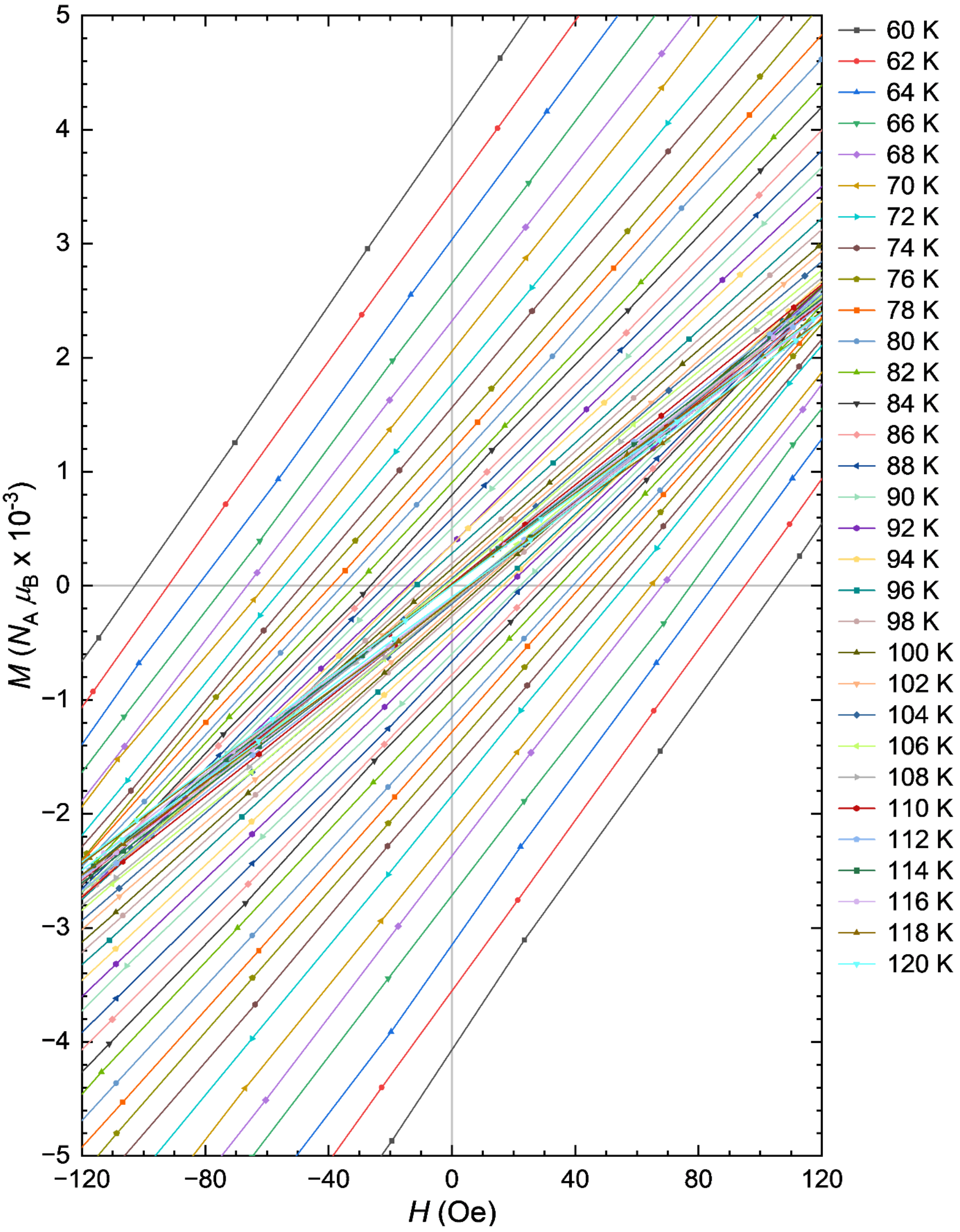

Fig. S32 | Magnetic hysteresis loops of 1-Dy. Temperatures from 60 to 120 K in 2 K increments and d.c. fields swept from –3 T to +3 T, zoomed in to ±120 Oe; sweep rate is 22 Oe s^{-1}.

Table S5 | Best fit parameters to the generalised Debye model for 1-Dy in zero d.c. field

T	τ_{Debye}	$\tau_{\text{Debye}}^{\text{err}}$	χ_S	χ_S^{err}	χ_T	χ_T^{err}	α	α^{err}	$\langle \ln \tau \rangle$	$\sigma_{\langle \ln \tau \rangle}$
(K)	(s)		(emu/mol)		(emu/mol)				ln (s)	
					**Waveform Measurements**					
2	78.3	4.08	7.13E–1	4.77E–2	4.81	7.54E–2	3.16E–1	1.96E–2	4.3603	1.9319
4	69.8	4.50	4.75E–1	3.90E–2	3.07	5.66E–2	3.54E–1	2.30E–2	4.2450	2.1414
5.5	63.4	3.72	3.64E–1	2.40E–2	2.04	3.28E–2	3.58E–1	2.11E–2	4.1496	2.1664
7.5	58.6	3.72	3.19E–1	2.28E–2	1.73	2.95E–2	3.59E–1	2.31E–2	4.0706	2.1741
10	49.6	2.87	2.51E–1	1.61E–2	1.26	1.88E–2	3.52E–1	2.19E–2	3.9035	2.1292
14	38.7	2.46	1.99E–1	1.46E–2	9.38E–1	1.46E–2	3.37E–1	2.55E–2	3.6550	2.0508
20	24.7	1.94	1.51E–1	1.47E–2	6.53E–1	1.08E–2	3.38E–1	3.21E–2	3.2051	2.0538
24	18.4	1.80	1.30E–1	1.66E–2	5.51E–1	9.88E–3	3.43E–1	3.84E–2	2.9115	2.0790
					**a.c. Measurements**					
52	2.09	1.23E–1	5.19E–2	2.53E–4	2.32E–1	5.54E–3	2.26E–1	9.94E–3	0.7358	1.4832
55	1.76	7.83E–2	4.98E–2	2.17E–4	2.18E–1	3.90E–3	2.23E–1	8.33E–3	0.5648	1.4696
58	1.50	5.09E–2	4.80E–2	1.86E–4	2.05E–1	2.80E–3	2.15E–1	7.05E–3	0.4056	1.4308
61	1.38	3.76E–2	4.62E–2	1.60E–4	1.99E–1	2.19E–3	2.15E–1	5.94E–3	0.3191	1.4324
64	1.16	2.58E–2	4.48E–2	1.48E–4	1.87E–1	1.67E–3	2.05E–1	5.44E–3	0.1472	1.3846
67	1.12	2.54E–2	4.34E–2	1.48E–4	1.84E–1	1.64E–3	2.19E–1	5.41E–3	0.1109	1.4524
70	9.63E–1	1.67E–2	4.20E–2	1.25E–4	1.75E–1	1.19E–3	2.14E–1	4.48E–3	–0.0377	1.4254
73	8.25E–1	1.80E–2	4.08E–2	1.77E–4	1.67E–1	1.44E–3	2.01E–1	6.29E–3	–0.1925	1.3666

76	7.34E–1	1.01E–2	3.96E–2	1.18E–4	1.62E–1	8.67E–4	2.08E–1	4.09E–3	–0.3093	1.3977
79	6.79E–1	1.12E–2	3.86E–2	1.45E–4	1.59E–1	1.01E–3	2.12E–1	4.98E–3	–0.3872	1.4181
82	6.05E–1	1.10E–2	3.76E–2	1.69E–4	1.54E–1	1.07E–3	2.11E–1	5.75E–3	–0.5029	1.4131
85	5.25E–1	9.73E–3	3.67E–2	1.78E–4	1.50E–1	1.02E–3	2.23E–1	5.90E–3	–0.6447	1.4700
88	4.55E–1	8.51E–3	3.54E–2	1.92E–4	1.46E–1	9.88E–4	2.24E–1	6.21E–3	–0.7869	1.4725
91	4.05E–1	5.49E–3	3.48E–2	1.46E–4	1.44E–1	6.96E–4	2.27E–1	4.59E–3	–0.9028	1.4909
94	3.29E–1	4.66E–3	3.40E–2	1.65E–4	1.40E–1	6.86E–4	2.33E–1	4.98E–3	–1.1116	1.5178
97	2.74E–1	4.14E–3	3.31E–2	1.91E–4	1.39E–1	7.06E–4	2.36E–1	5.47E–3	–1.2956	1.5341
100	2.08E–1	2.39E–3	3.25E–2	1.59E–4	1.36E–1	5.02E–4	2.40E–1	4.32E–3	–1.5697	1.5508
103	1.55E–1	1.76E–3	3.20E–2	1.72E–4	1.34E–1	4.64E–4	2.45E–1	4.40E–3	–1.8671	1.5769
106	1.07E–1	1.11E–3	3.15E–2	1.74E–4	1.31E–1	3.89E–4	2.39E–1	4.22E–3	–2.2348	1.5465
109	6.86E–2	9.68E–4	3.10E–2	2.66E–4	1.27E–1	4.77E–4	2.21E–1	6.10E–3	–2.6796	1.4589
112	4.30E–2	5.48E–4	3.07E–2	2.57E–4	1.24E–1	3.79E–4	2.23E–1	5.57E–3	–3.1476	1.4683
115	2.62E–2	3.49E–4	3.09E–2	2.92E–4	1.20E–1	3.52E–4	2.00E–1	6.10E–3	–3.6415	1.3594
118	1.56E–2	1.84E–4	3.02E–2	2.78E–4	1.17E–1	2.76E–4	2.01E–1	5.41E–3	–4.1635	1.3628
121	9.30E–3	1.51E–4	2.98E–2	4.13E–4	1.14E–1	3.38E–4	1.92E–1	7.54E–3	–4.6778	1.3223
124	5.63E–3	8.19E–5	3.01E–2	3.97E–4	1.11E–1	2.70E–4	1.76E–1	6.90E–3	–5.1789	1.2460
127	3.46E–3	4.76E–5	2.90E–2	4.03E–4	1.10E–1	3.31E–4	1.90E–1	6.81E–3	–5.6662	1.3110
130	2.12E–3	3.08E–5	2.98E–2	4.66E–4	1.07E–1	2.97E–4	1.68E–1	7.34E–3	–6.1543	1.2099
133	1.30E–3	1.94E–5	2.93E–2	5.25E–4	1.04E–1	2.54E–4	1.60E–1	7.43E–3	–6.6469	1.1707
136	8.16E–4	1.46E–5	2.93E–2	6.79E–4	1.02E–1	2.48E–4	1.45E–1	8.63E–3	–7.1109	1.0992

139	5.29E–4	9.69E–6	2.95E–2	7.33E–4	9.95E–2	1.97E–4	1.34E–1	8.22E–3	–7.5449	1.0485
142	3.58E–4	8.09E–6	3.12E–2	9.33E–4	9.74E–2	1.83E–4	1.11E–1	9.46E–3	–7.9363	0.9332
145	2.39E–4	7.95E–6	3.19E–2	1.37E–3	9.54E–2	1.92E–4	1.04E–1	1.19E–2	–8.3399	0.9003
148	1.60E–4	9.97E–6	3.19E–2	2.56E–3	9.34E–2	2.08E–4	8.98E–2	1.78E–2	–8.7406	0.8256
151	1.22E–4	9.52E–6	3.38E–2	3.27E–3	9.14E–2	1.83E–4	4.68E–2	2.02E–2	–9.0145	0.5751

Table S6 | Best fit parameters to the generalised Debye model for 5%Dy@1-Y in zero d.c. field

T	τ_{Debye}	$\tau_{\mathrm{Debye}}^{\mathrm{err}}$	χ_S	χ_S^{err}	χ_T	χ_T^{err}	α	α^{err}	$\langle \ln \tau \rangle$	$\sigma_{\langle \ln \tau \rangle}$
(K)	(s)		(cm^3/mol)		(cm^3/mol)				ln (s)	
**Waveform Measurements**										
4	928	400	7.26E–1	2.71E–2	2.08	1.86E–1	0.451	6.11E–2	6.83	2.76
7.5	1271	776	4.35E–1	2.81E–2	1.72	2.33E–1	0.494	6.69E–2	7.15	3.09
14	141	25.4	2.74E–1	1.90E–2	9.10E–1	3.79E–2	0.368	5.54E–2	4.95	2.22
24	28.6	4.97	1.88E–1	2.21E–2	5.59E–1	1.70E–2	0.350	6.75E–2	3.35	2.12
**a.c. Susceptibility Measurements**										
55	2.53	2.11	1.09E–1	3.05E–3	3.35E–1	7.14E–2	0.395	8.55E–2	0.929	2.38
59	1.73	0.763	1.08E–1	1.82E–3	2.80E–1	2.92E–2	0.379	5.72E–2	0.545	2.29
63	1.2	0.394	1.07E–1	1.60E–3	2.57E–1	1.93E–2	0.354	5.19E–2	0.182	2.15
67	0.608	0.127	1.07E–1	1.85E–3	2.22E–1	1.12E–2	0.248	6.07E–2	–0.498	1.59
71	1.86	0.682	1.04E–1	1.49E–3	2.91E–1	2.67E–2	0.373	4.56E–2	0.621	2.25
75	0.695	0.255	1.04E–1	2.05E–3	2.29E–1	2.10E–2	0.282	7.47E–2	–0.364	1.76
79	0.711	0.175	1.03E–1	1.69E–3	2.24E–1	1.23E–2	0.321	5.38E–2	–0.341	1.96
83	0.448	9.51E–2	9.98E–2	1.89E–3	2.33E–1	1.07E–2	0.350	4.80E–2	–0.803	2.12
87	0.42	7.60E–2	1.00E–1	1.50E–3	2.20E–1	8.12E–3	0.350	4.16E–2	–0.867	2.12
91	0.38	7.72E–2	1.05E–1	2.43E–3	2.12E–1	9.53E–3	0.262	6.66E–2	–0.969	1.66
95	0.219	4.47E–2	1.08E–1	3.79E–3	2.07E–1	9.07E–3	0.195	9.05E–2	–1.52	1.34
99	0.154	3.43E–2	1.07E–1	4.51E–3	2.08E–1	8.48E–3	0.299	8.38E–2	–1.87	1.85

103	0.142	3.34E–2	1.03E–1	4.40E–3	2.15E–1	9.55E–3	0.308	8.28E–2	–1.95	1.89
107	0.118	2.79E–2	1.09E–1	4.79E–3	2.04E–1	1.05E–2	0.144	0.117	–2.13	1.10
115	2.32E–2	5.02E–3	1.05E–1	4.88E–3	1.93E–1	5.93E–3	0.227	9.69E–2	–3.76	1.49
119	1.35E–2	4.07E–3	1.05E–1	7.29E–3	1.96E–1	7.97E–3	0.294	0.122	–4.30	1.82
123	8.70E–3	2.42E–3	9.63E–2	7.93E–3	2.08E–1	8.76E–3	0.401	9.22E–2	–4.74	2.42
127	6.86E–3	3.53E–3	8.46E–2	1.98E–2	2.24E–1	1.57E–2	0.569	0.114	–4.98	3.80

*Magnetic Data for **2-Dy***

The magnetic properties of **2-Dy** have been characterised by SQUID magnetometry. The room temperature molar magnetic susceptibility temperature product (χT) is 14.2 cm^3 K mol^{-1} (Fig. S33), in excellent agreement with the free-ion value of 14.17 cm^3 K mol^{-1} and the CASSCF-SO calculated value. (13.66 cm^3 K mol^{-1}). The value of χT decreases until *ca.* 20 K and then rapidly decreases below 6 K, indicating magnetic blocking. Magnetic blocking is confirmed by the zero-field cooled (ZFC) and field-cooled (FC) susceptibility: the ZFC susceptibility peaks at 9 K (T_{peak}) and ZFC and field-cooled (FC) traces diverge at 19 K (T_{irrev}; Figs. S34 and S35). This irreversible point occurs at a much lower temperature than for **1-Dy** (47 K), even when using a faster temperature sweep rate. The magnetisation saturates at 5.34 N_A μ_B, indicating a majority m_J = ± 15/2 ground state (Fig. S36). Hysteresis curves are closed around zero-field at 2 and 4 K (Fig. S37).

Slow magnetisation reversal in **2-Dy** was investigated by a.c. susceptibility, with peaks being observed between 1.8 and 45 K (Fig. S38–S40). Fitting of the data to the Generalised Debye model in CC-FIT2 gave α values of 0.0398–0.214. The temperature dependence of the magnetisation reversal times was fit to a sum of Orbach, power-law Raman and QTM terms: U_{eff} = 597 ± 40 K (415 ± 28 cm^{-1}), τ_0 = $10^{-9.0\pm0.4}$ s, $C = 10^{-2.24\pm0.08}$ s^{-1} K^{-n}, n = 3.37±0.06, $\tau_{QTM} = 10^{-1.900\pm0.008}$ s (Fig. S41). While the CASSCF results suggest magnetic reversal via the second excited state (950 K, 665 cm^{-1}, $g_{x,y,}$ ~ 0.2, g_z at 14° from the ground state, Table S7), the Orbach barrier indicates reversal occurs via the first excited state (91.5% m_J = 13/2) at 513 K (359 cm^{-1}). While U_{eff} is still large, it is significantly reduced compared to **1-Dy** because of the negatively charged allyl group coordinating to the Dy centre and the more bent N–Dy–N angle. The QTM rate is approximately four orders or magnitude greater than for

1-Dy because of the less axial crystal field and the Raman rates are also notably faster.

Magnetic measurements were performed on a 35.7 mg microcrystalline powder sample of **2-Dy** with 21.8 mg of eicosane, with the sample prepared as for **1-Dy** and **5%Dy@1-Y**. Equilibrium susceptibility measurements on **2-Dy** in 0.1 T d.c. field were performed between 300 and 1.8 K in temperature settle mode, with cooling rates of 5 K min^{-1} from 300–100 K, 2 K min^{-1} from 100–10 K and 1 K min^{-1} from 10–1.8 K. For temperatures of 50–25 K and 22–1.8 K, wait times of 4 and 20 min respectively were used at each temperature to attempt to measure the equilibrium susceptibility. Measurements were performed using d.c. mode with a 40 mm scan length and 6 s scan time.

A zero-field cooled (ZFC) sample of **2-Dy** was prepared by waiting for 30 min at 50 K in zero-field, cooling to 10 K at 5 K min^{-1}, holding for 5 min, cooling to 2 K at 1 K min^{-1} and holding for 30 min before switching on the field to 0.1 T. The ZFC susceptibility was measured on warming, then the field-cooled (FC) susceptibility was measured on cooling and warming. Measurements were performed in continuous temperature sweep mode with a sweep rate of 0.9 K min^{-1} and using d.c. mode with a 40 mm scan length and 6 s scan time.

Magnetisation vs. field curves (0–7 T) were recorded for **2-Dy** at 2 and 4 K, using VSM mode with 5 mm vibration amplitude and 2 s averaging time. Hysteresis measurements were performed on **2-Dy** between ± 7 T at 2 and 4 K. VSM mode with a 2 mm vibration amplitude and 4 s (2 K) or 5 s (4 K) averaging time was used. The field was swept continually, with sweep rates of 22 Oe s^{-1} for $|H| \leq 1$ T (points every ~250 Oe), 54 Oe s^{-1} for $1 < |H| \leq 2$ T (points every ~1000 Oe) and 91 Oe s^{-1} for $2 < |H| \leq 7$ T (points every ~1250 Oe).

Alternating frequency (a.c.) susceptibility measurements were recorded on **2-Dy** at temperatures between 1.8–45 K in zero d.c. field. Measurements were performed using 5 frequencies per decade between 0.1–1000 Hz with an oscillating field of 2 Oe. Averages were performed for 2 s or for 10 cycles, whichever was longer. A.c. data were fit to the Generalised Debye model in CC-FIT2 to extract magnetisation reversal rates and distributions. The resulting rates were fit to a sum of Orbach, Raman and QTM process in CC-FIT2.

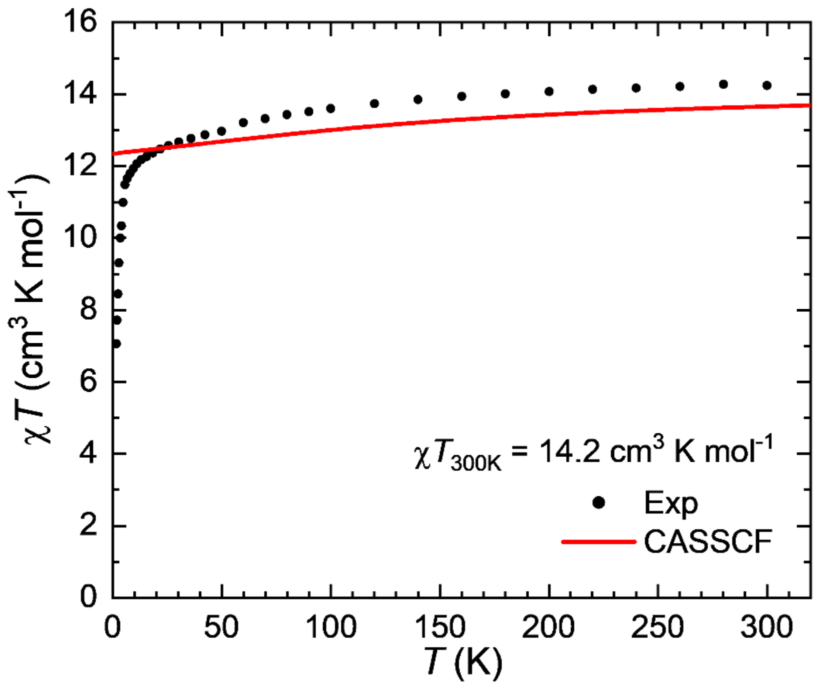

Fig. S33 | Temperature dependence of the molar magnetic susceptibility χT product for 2-Dy. Measured under a 0.1 T applied magnetic field on cooling (black circles) with CASSCF-SO calculated trace (red line).

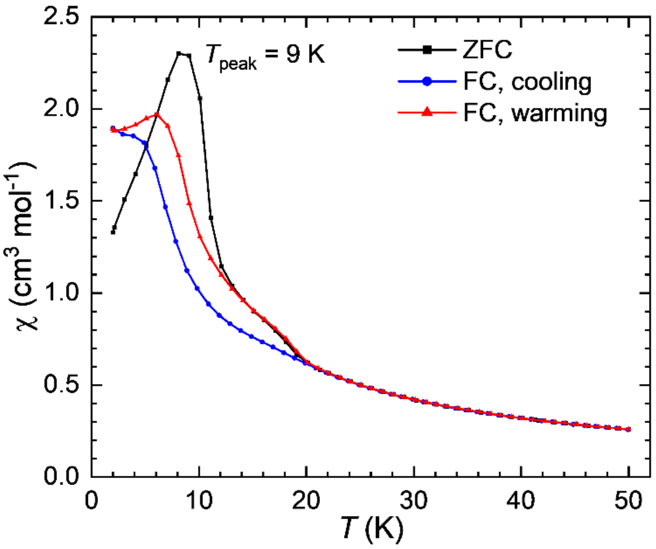

Fig. S34 | Magnetic susceptibility (χ) vs. temperature for 2-Dy. Measured under a 0.1 T applied magnetic field on warming after cooling in zero-field (ZFC, black), on cooling in field (FC cooling, blue) and on warming in field (FC warming, red). Sweep rate is 0.9 K min^{-1}.

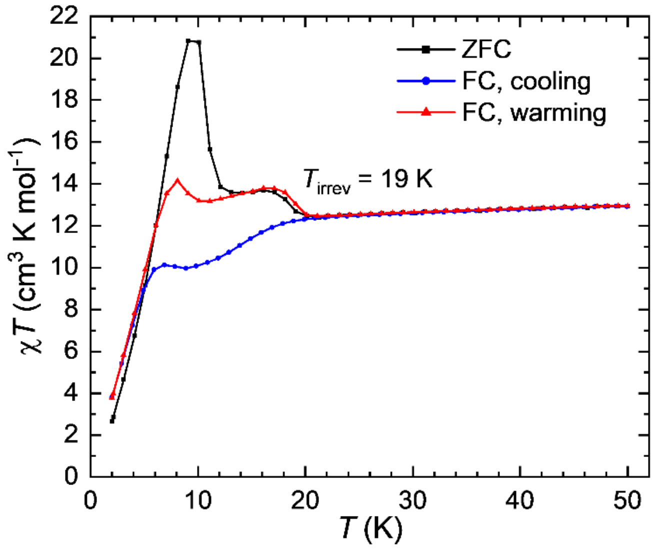

Fig. S35 | Magnetic susceptibility temperature product (χ*T*) vs. temperature for 2-Dy. Measured under a 0.1 T applied magnetic field on warming after cooling in zero-field (ZFC, black), on cooling in field (FC cooling, blue) and on warming in field (FC warming, red). Sweep rate is 0.9 K min^{-1}.

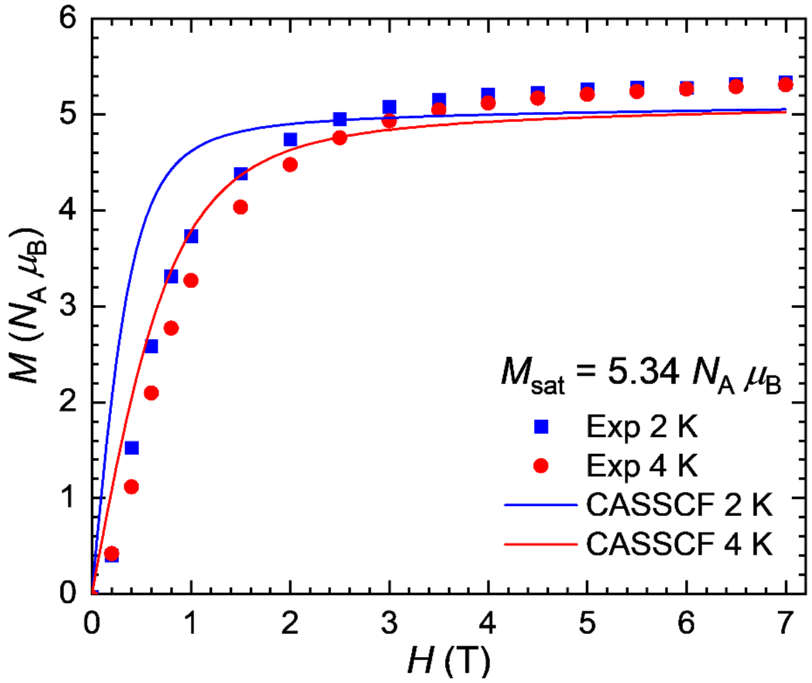

Fig. S36 | Magnetisation vs. field curves for 2-Dy at 2 and 4 K. Solid lines indicate CASSCF-SO calculated curves.

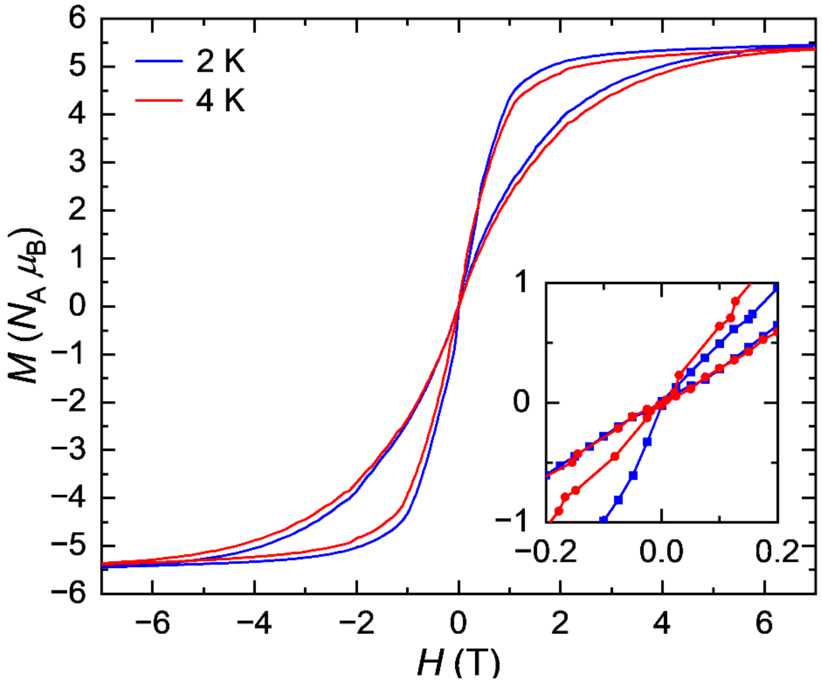

Fig. S37 | Hysteresis loops of 2-Dy at 2 and 4 K and –7 T to +7 T. Sweep rate is 22 Oe s^{-1} for |H| ≤ 1 T, 54 Oe s^{-1} for 1 < |H| ≤ 2 T and 91 Oe s^{-1} for 2 < |H| ≤ 7 T. Inset shows closed loops around zero-field.

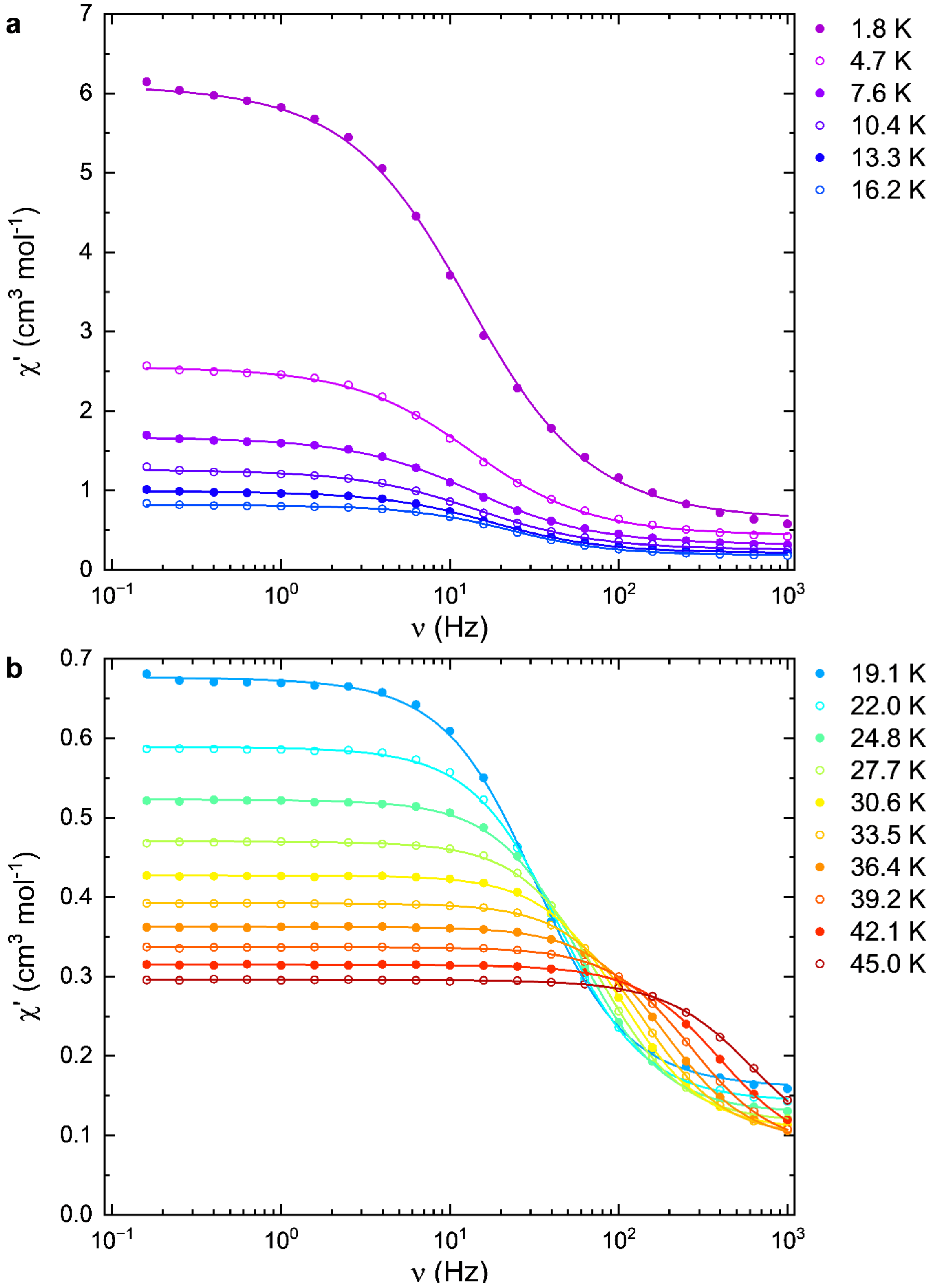

Fig. S38 | Temperature-dependence of in-phase (χ′) a.c. susceptibility data for 2-Dy. a, 1.8–16.2 K. **b**, 19.1–45.0 K. All data fitted in zero d.c. field, using the generalised Debye model in CC-FIT2.

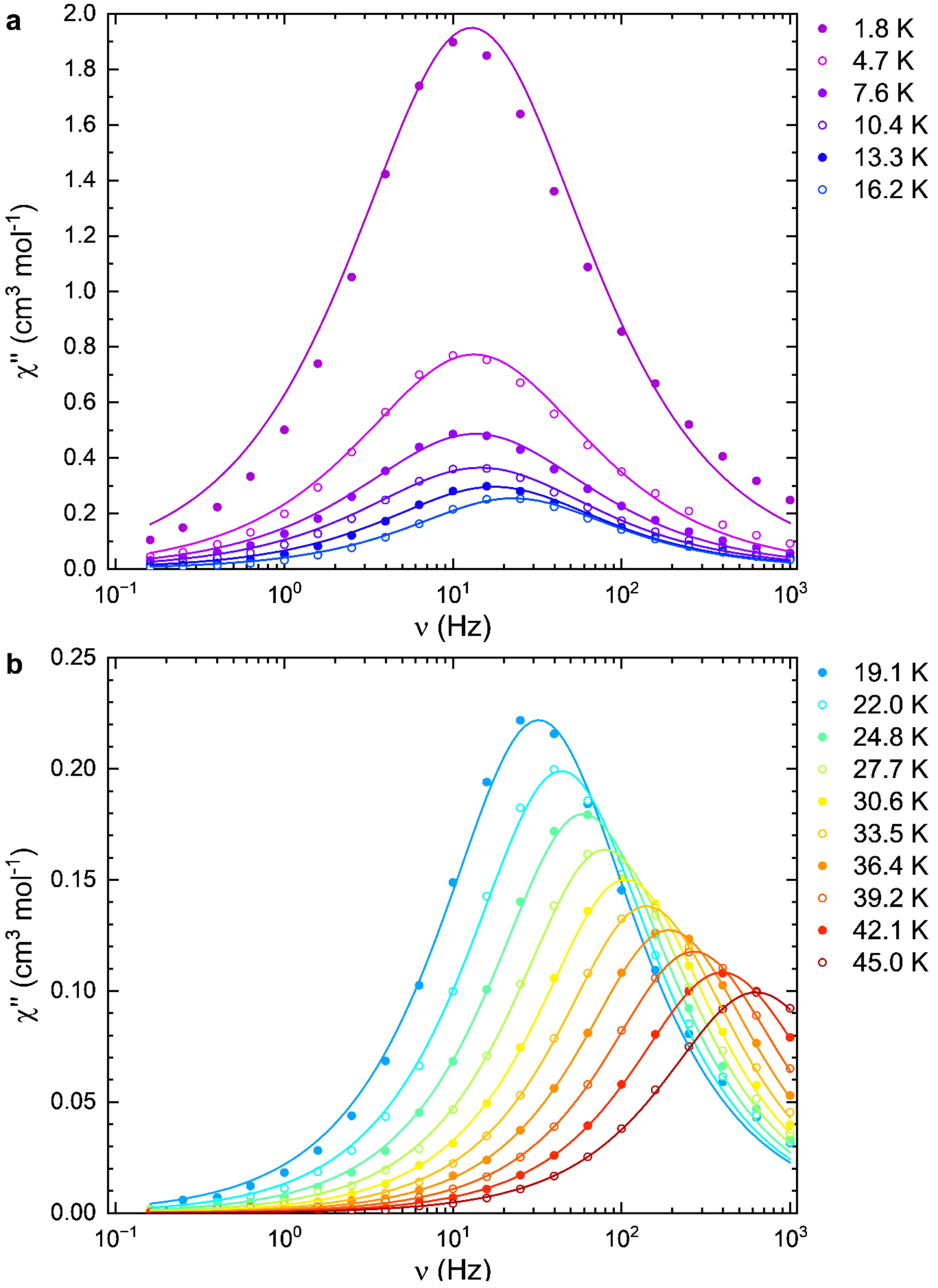

Fig. S39 | Temperature-dependence of out-of-phase (χ″) a.c. magnetic susceptibility for 2-Dy. a, 1.8–16.2 K. **b**, 19.1–45.0 K. All data fitted in zero d.c. field, using the generalised Debye model in CC-FIT2.

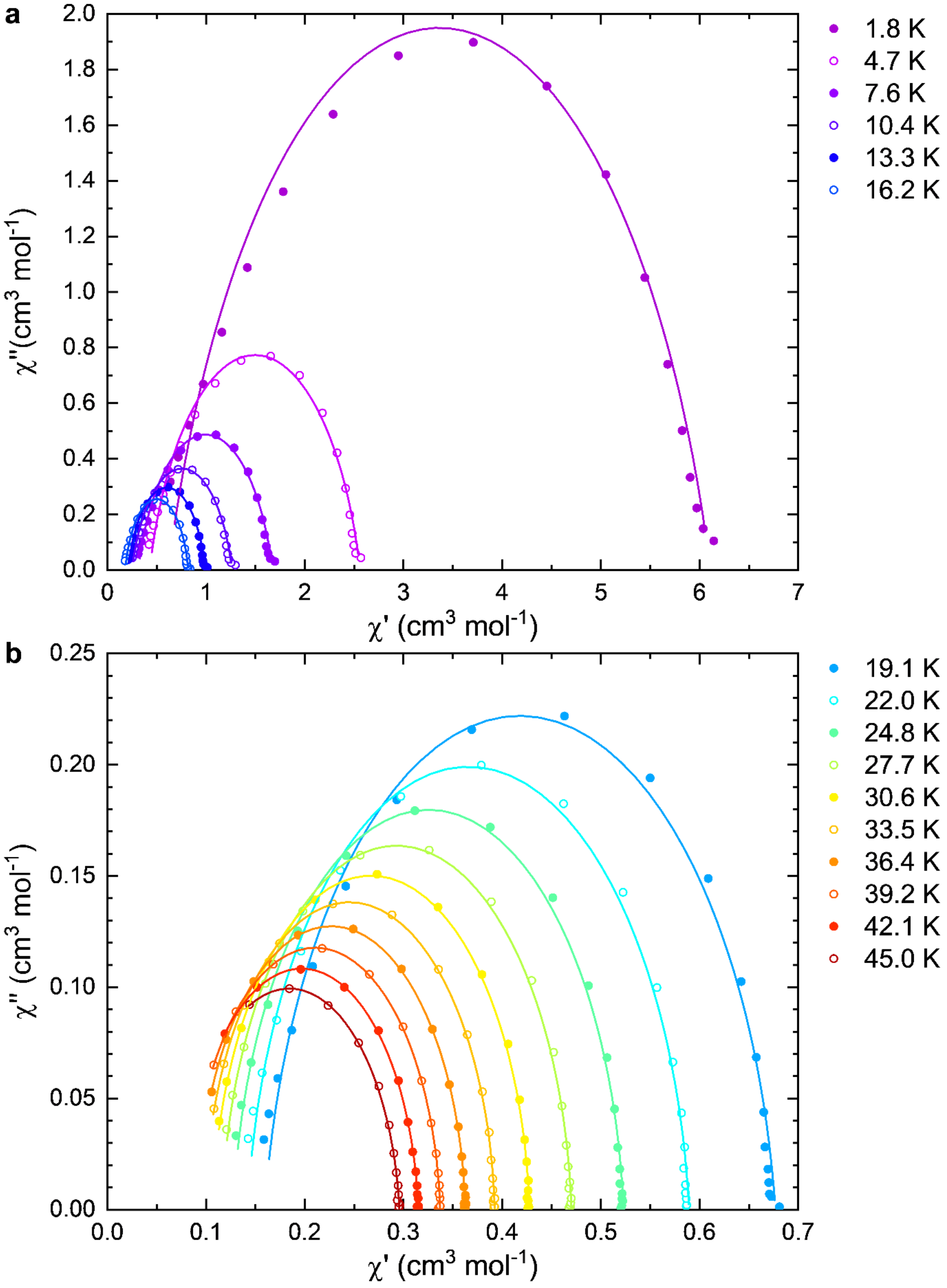

Fig. S40 | Cole-Cole plot for to show temperature-dependence of ın-phase (χ′) and out-of-phase (χ″) a.c. susceptibilities for 2-Dy. a, 1.8–16.2 K. **b**, 19.1–45.0 K. All data fitted in zero d.c. field, using the generalised Debye model in CC-FIT2.

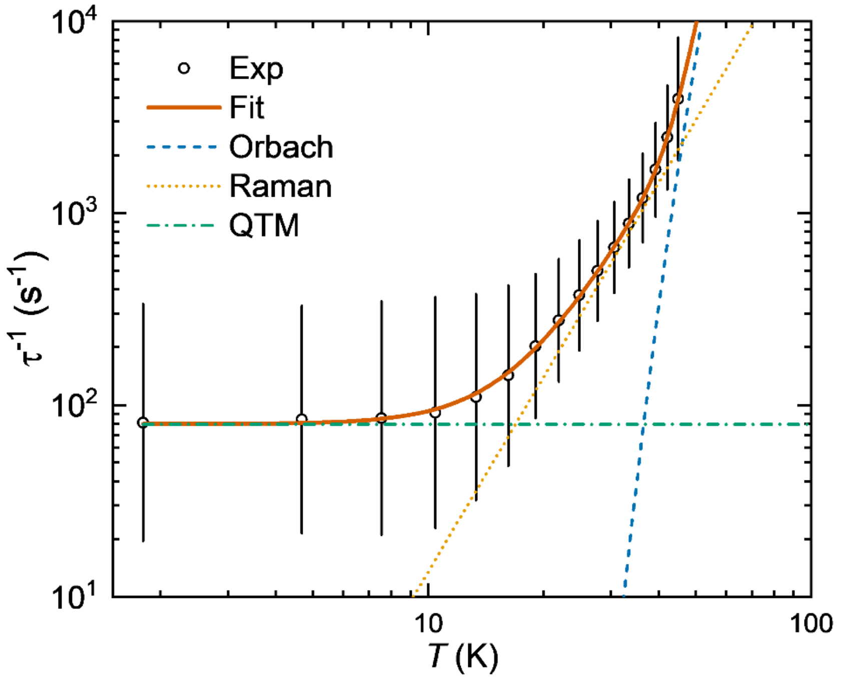

Fig. S41 | Fitting of magnetisation reversal rates for 2-Dy. Under zero d.c. field, fitted using CC-FIT2. Orbach, Raman and QTM components shown. Error bars represent 1 estimated standard deviation of the distribution of rates.

Table S7 | Best fit parameters to the generalised Debye model for 2-Dy in zero d.c. field

T	τ_{debye}	τ_{Debye}^{err}	χ_S	χ_S^{err}	χ_T	χ_T^{err}	α	α^{err}	$\langle \ln \tau \rangle$	$\sigma_{\langle \ln \tau \rangle}$
(K)	(s)		(emu/mol)		(emu/mol)				ln (s)	
1.80	1.24E–2	2.93E–4	6.18E–1	3.55E–2	6.11	3.51E–2	2.14E–1	1.07E–2	–4.3941	1.4257
4.68	1.19E–2	2.26E–4	4.27E–1	1.12E–2	2.56	1.10E–2	2.02E–1	8.71E–3	–4.4299	1.3684
7.56	1.17E–2	2.26E–4	3.13E–1	7.25E–3	1.67	7.04E–3	2.09E–1	8.74E–3	–4.4475	1.4020
10.44	1.10E–2	2.35E–4	2.49E–1	6.11E–3	1.26	5.78E–3	2.06E–1	9.77E–3	–4.5125	1.3872
13.32	9.10E–3	1.69E–4	2.10E–1	4.38E–3	9.92E–1	3.87E–3	1.75E–1	8.96E–3	–4.6996	1.2405
16.20	7.02E–3	1.21E–4	1.82E–1	3.59E–3	8.19E–1	2.90E–3	1.41E–1	8.74E–3	–4.9591	1.0822
19.08	4.94E–3	6.38E–5	1.59E–1	2.46E–3	6.77E–1	1.77E–3	9.72E–2	7.04E–3	–5.3110	0.8641
21.96	3.62E–3	3.78E–5	1.42E–1	1.87E–3	5.89E–1	1.22E–3	7.40E–2	5.90E–3	–5.6217	0.7393
24.84	2.68E–3	2.12E–5	1.28E–1	1.35E–3	5.23E–1	7.90E–4	6.04E–2	4.56E–3	–5.9218	0.6608
27.72	2.00E–3	1.26E–5	1.16E–1	1.04E–3	4.70E–1	5.44E–4	5.04E–2	3.68E–3	–6.2135	0.5984
30.60	1.51E–3	7.80E–6	1.07E–1	8.38E–4	4.27E–1	3.90E–4	4.23E–2	3.06E–3	–6.4967	0.5449
33.48	1.13E–3	4.40E–6	9.81E–2	6.21E–4	3.92E–1	2.53E–4	3.98E–2	2.30E–3	–6.7835	0.5274
36.36	8.34E–4	3.25E–6	9.09E–2	6.21E–4	3.63E–1	2.14E–4	4.08E–2	2.27E–3	–7.0893	0.5346
39.24	5.94E–4	2.21E–6	8.43E–2	5.93E–4	3.37E–1	1.65E–4	4.49E–2	2.07E–3	–7.4291	0.5628
42.12	4.03E–4	2.10E–6	7.89E–2	8.20E–4	3.15E–1	1.69E–4	5.43E–2	2.60E–3	–7.8165	0.6235
45.00	2.54E–4	4.00E–6	7.28E–2	2.38E–3	2.96E–1	3.09E–4	7.35E–2	6.25E–3	–8.2789	0.7364

9. Ab initio spin dynamics

Table S8 | CASSCF-SO-calculated electronic structure of 1-Dy at the solid-state DFT optimised geometry of 1-Dy[a]

Energy (cm^{-1})	Energy (K)	g_x	g_y	g_z	Angle[b] (deg)	<J_z>	Wavefunction
0.00	0.00	0.00	0.00	19.99	0.00	±7.49	99.80% |±15/2>
523.92	748.58	0.00	0.00	16.99	0.46	±6.48	96.78% |±13/2>
1007.51	1439.52	0.04	0.05	14.05	3.54	±5.43	93.24% |±11/2>
1388.64	1984.07	0.41	0.42	11.32	14.27	±4.26	82.56% |±9/2> + 7.34% |±5/2>
1633.87	2334.46	0.39	1.10	9.00	32.93	±2.87	59.49% |±7/2> + 16.88% |±3/2> + 7.28% |±9/2> + 5.12% |±5/2>
1772.05	2531.88	4.02	6.67	7.80	82.89	±0.47	23.33% |±5/2> + 19.95% |±7/2> + 19.71% |±1/2> + 13.97% |∓1/2> + 9.99% |∓5/2>
1865.92	2666.01	1.16	2.53	15.99	86.08	±0.42	34.18% |±3/2> + 20.82% |∓5/2> + 15.99% |±5/2> + 15.12% |∓1/2>
1916.43	2738.17	0.22	0.43	19.59	86.90	±0.38	24.68% |±3/2> + 24.05% |∓1/2> + 17.63% |±1/2> + 10.72% |∓3/2> + 9.66% |∓5/2> + 6.28% |±5/2>

[a] Point charges used and only including 18 S = 5/2 spin-free states as used in spin-phonon calculations, in zero-field. Each row corresponds to a Kramers doublet. All data is directly from the CASSCF-SO calculation, except for the Wavefunction which is necessarily obtained by projection onto a model space of the $^6H_{15/2}$ term. [b] The angle between the g_z value of the excited Kramers doublet and the ground Kramers doublet

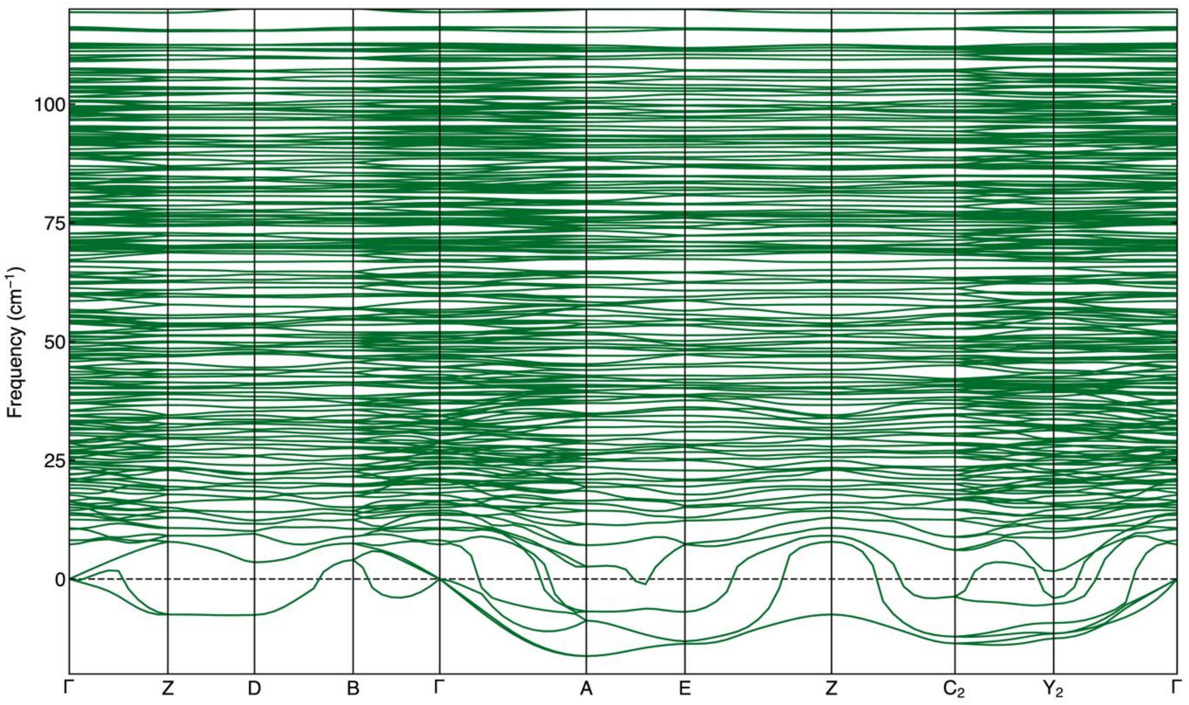

Fig. S42 | DFT-calculated low-energy phonon dispersion plot of 1-Dy.

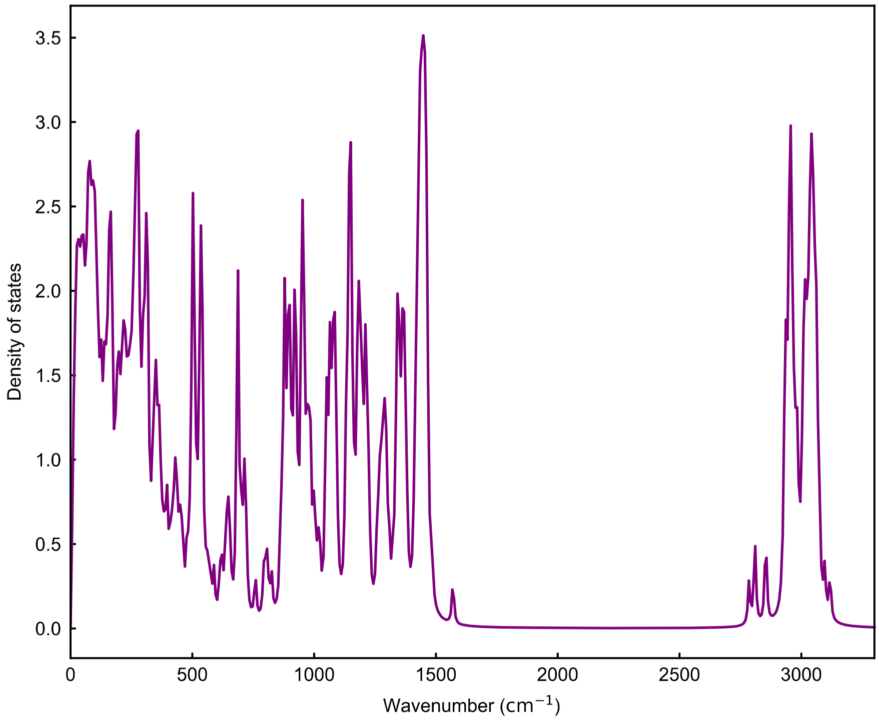

Fig. S43 | DFT-calculated density of states (DoS) of 1-Dy. Modes are broadened by anti-Lorentzian functions with a linewidth of 10 cm^{-1} and sampled on a 1×1×1 *q*-mesh.

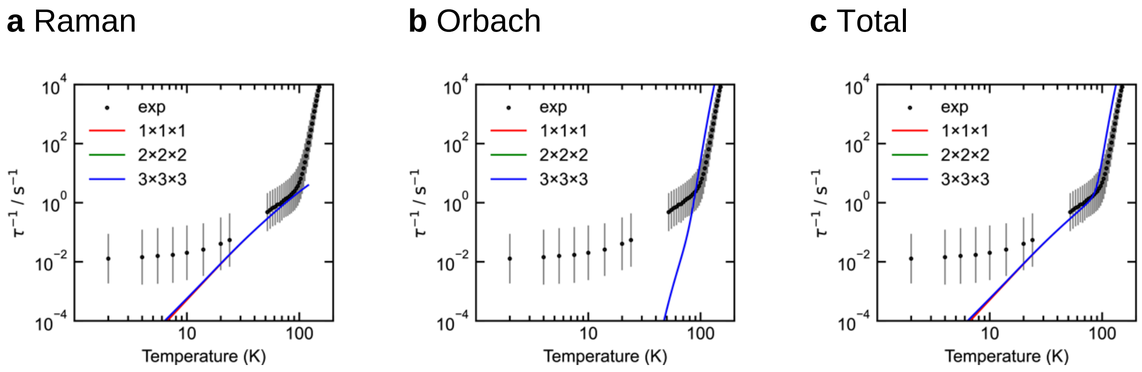

Fig. S44 | Comparison of experimental and calculated magnetic reversal rates for 1-Dy as a function of *q*-point mesh. a, Calculated Raman rates. **b**, Calculated Orbach rates. **c**, Total of calculated Raman and Orbach rates. Experimental magnetic reversal rates shown in black circles in each case. Fixed full-width-at-half-maximum linewidth Γ = 10 cm^{-1}. Calculations performed using solid-state phonon modes on a 1×1×1 (red), 2×2×2 (green) and 3×3×3 (blue) *q*-mesh. Imaginary modes are removed. Error bars correspond to one estimated standard deviation.

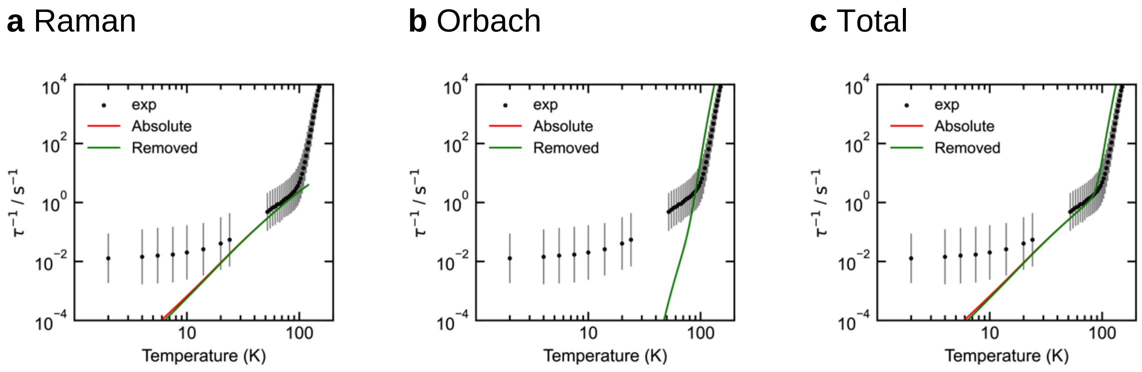

Fig. S45 | Comparison of experimental and calculated magnetic reversal rates for 1-Dy with and without imaginary phonon modes. a, Calculated Raman rates. **b**, Calculated Orbach rates. **c**, Total of calculated Raman and Orbach rates. Experimental magnetic reversal rates shown in black circles in each case. Fixed full-width-at-half-maximum linewidth Γ = 10 cm^{-1}. Calculations performed using solid-state phonon modes on a 3×3×3 *q*-mesh, with imaginary modes set to their absolute value

(red) and removed (green). Error bars correspond to one estimated standard deviation.

a Raman **b** Orbach **c** Total

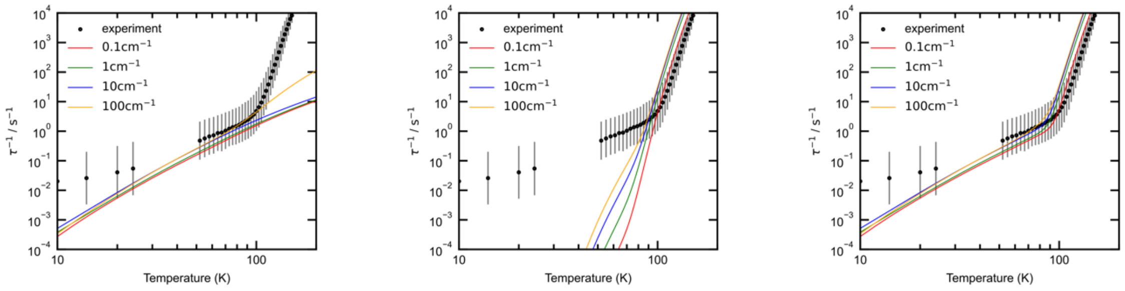

Fig. S46 | Comparison of experimental and calculated magnetic reversal rates for 1-Dy as a function of phonon linewidth. **a**, Calculated Raman rates. **b**, Calculated Orbach rates. **c**, Total of calculated Raman and Orbach rates. Experimental magnetic reversal rates shown in black circles in each case. Fixed full-width-at-half-maximum linewidth Γ = 0.1 cm^{-1} (red), 1 cm^{-1} (green), 10 cm^{-1} (blue) and 100 cm^{-1} (orange). Error bars correspond to one estimated standard deviation.

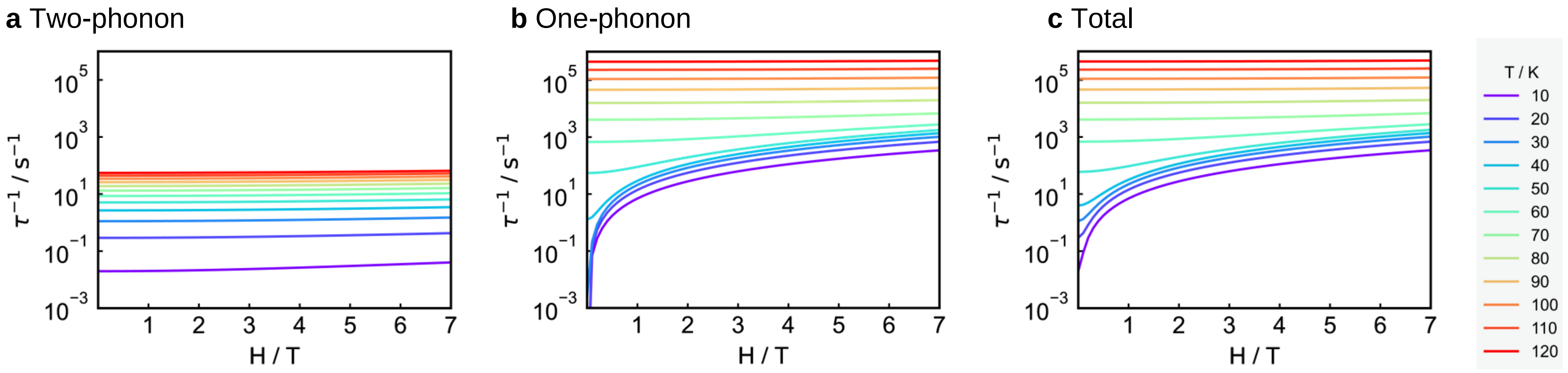

Fig. S47 | Calculated magnetic reversal rates for 1-Dy as a function of field. Aligned approximately perpendicular to the principal magnetic axis (θ = 89.4°, φ = 102.1°). **a**, Two-phonon rates. **b**, One-phonon rates. **c**, Total rates.

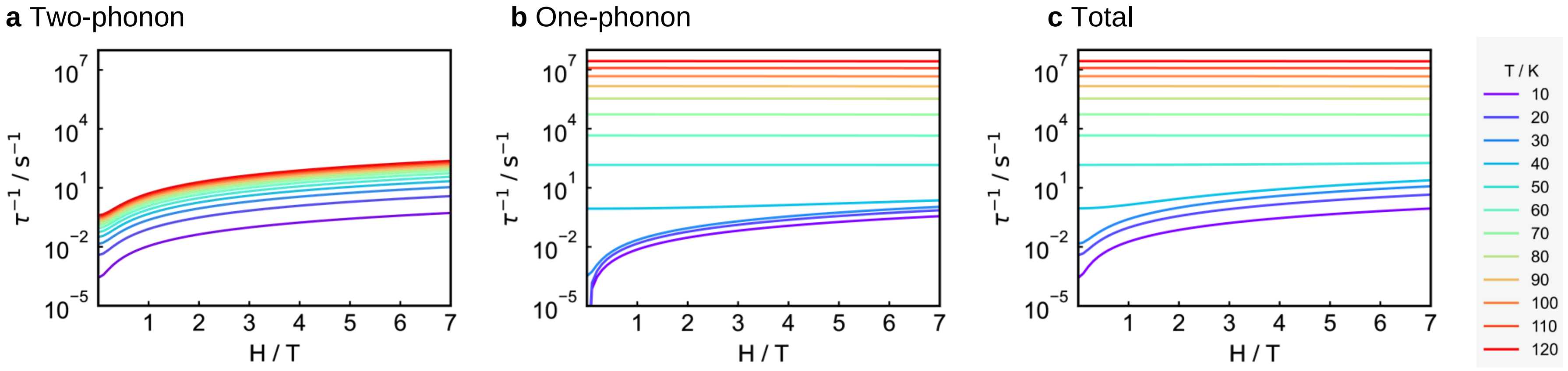

Fig. S48 | Calculated magnetic reversal rates for $[Dy(Cp^{ttt})_2][B(C_6F_5)_4]$ as a function of field. Aligned approximately perpendicular to the principal magnetic axis (θ = 89.4°, φ = 102.1°). **a**, Two-phonon rates. **b**, One-phonon rates. **c**, Total rates.

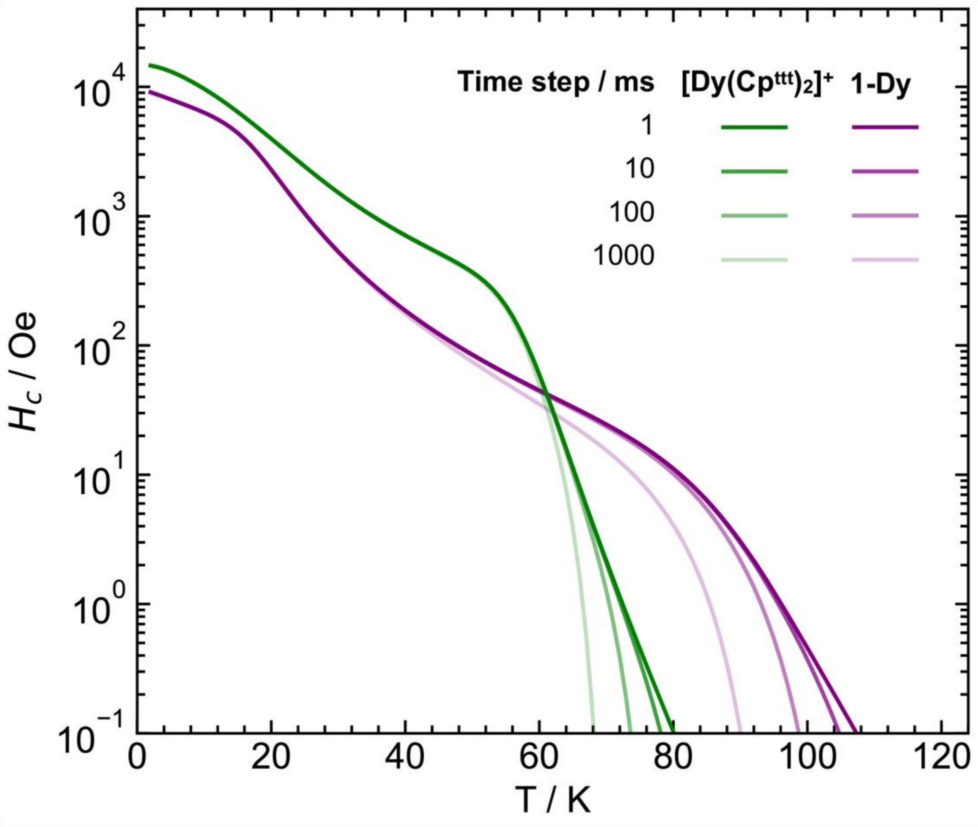

Fig. S49 | Calculated coercive field for 1-Dy and [Dy(Cp^{ttt})$_2$][B(C_6F_5)$_4$] as a function of temperature. Time steps of 1, 10, 100 and 1000 ms are shown.
